# Nucleotide composition of transposable elements likely contributes to AT/GC compositional homogeneity of teleost fish genomes

**DOI:** 10.1186/s13100-019-0195-y

**Published:** 2019-12-12

**Authors:** Radka Symonová, Alexander Suh

**Affiliations:** 1 Department of Biology, Faculty of Science, University of Hradec Králové, Hradec Králové, Czech Republic; 2 Department of Ecology and Genetics - Evolutionary Biology, Evolutionary Biology Centre (EBC), Science for Life Laboratory, Uppsala University, Uppsala, Sweden; 3 Present address: Department of Organismal Biology - Systematic Biology, Evolutionary Biology Centre (EBC), Science for Life Laboratory, Uppsala University, Uppsala, Sweden

**Keywords:** Teleost fish, Transposon, GC content, Genome evolution, Nucleotide composition

## Abstract

**Background:**

Teleost fish genome size has been repeatedly demonstrated to positively correlate with the proportion of transposable elements (TEs). This finding might have far-reaching implications for our understanding of the evolution of nucleotide composition across vertebrates. Genomes of fish and amphibians are GC homogenous, with non-teleost gars being the single exception identified to date, whereas birds and mammals are AT/GC heterogeneous. The exact reason for this phenomenon remains controversial. Since TEs make up significant proportions of genomes and can quickly accumulate across genomes, they can potentially influence the host genome with their own GC content (GC%). However, the GC% of fish TEs has so far been neglected.

**Results:**

The genomic proportion of TEs indeed correlates with genome size, although not as linearly as previously shown with fewer genomes, and GC% negatively correlates with genome size in the 33 fish genome assemblies analysed here (excluding salmonids). GC% of fish TE consensus sequences positively correlates with the corresponding genomic GC% in 29 species tested. Likewise, the GC contents of the entire repetitive vs. non-repetitive genomic fractions correlate positively in 54 fish species in Ensembl. However, among these fish species, there is also a wide variation in GC% between the main groups of TEs. Class II DNA transposons, predominant TEs in fish genomes, are significantly GC-poorer than Class I retrotransposons. The AT/GC heterogeneous gar genome contains fewer Class II TEs, a situation similar to fugu with its extremely compact and also GC-enriched but AT/GC homogenous genome.

**Conclusion:**

Our results reveal a previously overlooked correlation between GC% of fish genomes and their TEs. This applies to both TE consensus sequences as well as the entire repetitive genomic fraction. On the other hand, there is a wide variation in GC% across fish TE groups. These results raise the question whether GC% of TEs evolves independently of GC% of the host genome or whether it is driven by TE localization in the host genome. Answering these questions will help to understand how genomic GC% is shaped over time. Long-term accumulation of GC-poor(er) Class II DNA transposons might indeed have influenced AT/GC homogenization of fish genomes and requires further investigation.

## Background

Nucleotide composition is a fundamental property of genomes with a strong influence on gene function and regulation [1]. Hence, GC content of a genome (GC_G_), i.e., the molar ratio of guanine (G) and cytosine (C) in DNA, is one of the main parameters used to describe nucleotide composition and is frequently related to genome size [1]. For practical reasons, genomes can be segmented in five types of regions called isochores according to their GC percentage (GC%). Two “light” isochores with the lowest GC%, i.e., L1 with approx. 34–36% of GC and L2 approx. 37–40% of GC; as well as three “heavy” isochores, i.e., H1 with approx. 41–45% of GC, H2 46–52% and the “heaviest” H3 with > 53% of GC [2]. In this regard, fish and amphibian genomes are overall AT/GC homogenous because they contain only the GC-poor(er) isochores with a substantially narrower range of GC%, i.e., usually only two neighbouring ones such as L1 and L2 or L2 and H1. On the other hand, avian and mammalian genomes contain all five isochores and their broad range of GC% results in overall GC heterogeneity [2].

An increasing number of recent studies in fish has shown a clear positive correlation between genome size and percentage of TEs, and that TEs are ubiquitous and present in large numbers, e.g., refs. [3–6]. One of these studies [7] documented a surprisingly linear correlation between genome size and TE content in four teleost fish species. A clear but not strictly linear correlation between the percentage of TEs and genome size was identified in a larger dataset of 19 ray-finned and two lobe-finned fish species ([3]; including the four genomes analysed by ref. [7]). The so far most extensive (but still unpublished) study on fish TEs by ref. [5] using in silico explorations of TE activity, diversity and abundance across 74 teleost fish genomes showed that the total genomic TE abundances reflect variation in their host genome size.

Moreover, TEs can be very different in copy numbers and composition [3, 4, 8, 9], which would imply that accumulation or turnover of TE numbers/composition could change genomic GC content (GC_G_) because of the TEs’ own GC content (GC_TE_). There are major quantitative and qualitative differences in TEs among vertebrates: Class II DNA transposons are the most abundant group in fish genomes, whereas in avian and mammalian genomes Class I retrotransposons are the most abundant group while DNA transposons are substantially less numerous [3–5, 8, 9]. Hence, the GC_TE_ of different mobilomes, i.e., the sum of TEs within a genome, may potentially result in different overall GC_G_ organization in fish when compared with birds and mammals. However, the characteristics of GC_TE_ remains understudied in general, particularly in fish. This is despite the fact that TEs make up 6–55% of the total base pairs of fish genomes, and that TEs are clearly depleted in compact and GC-rich genomes (*Takifugu flavidus* [9, 10], *Tetraodon nigroviridis* [11, 12]) while they are massively represented in large and GC-poor genomes such zebrafish (*Danio rerio* [13]) and cod (*Gadus morhua* [14]).

The currently known main features of fish mobilomes can be summarized as follows: i. DNA transposons are the predominant group of TEs in fish; ii. the diversity of TE families is generally high in fish; iii. many TEs show recent activity in fish genomes; and iv. the total genomic abundances of TEs reflect the variation in genome size [3–5, 15]. Since the dynamics of genome size variation can be largely explained by TEs in many eukaryotes [16, 17] and GC_G_ is negatively linked to genome size in some organisms [1], these findings in fish raise crucial questions about potential roles of TEs in shaping GC_G_: i. Do TEs have a different GC% than the non-TE regions of the host genome? ii. Do new TE insertions lead to a decrease in GC% in adjacent regions of the host genome because of TE silencing through cytosine methylation? Methylcytosine frequently undergoes spontaneous deamination resulting in point mutation to thymine [18]. iii. Do TEs change local recombination rates (negatively if TEs are heterochromatinized or positively if they contain motifs attracting the recombination machinery [19, 20]) and hence influence the GC_G_ as discussed below? These factors all may contribute to the overall nucleotide compositional landscape, i.e., the heterogeneous organization in birds and mammals in comparison with the homogeneous organization in fish and amphibians. Such manifold effects of TEs might be particularly pronounced in species where TEs comprise a substantial genomic fraction, e.g., zebrafish (*D. rerio*) [13].

Both the local GC_G_ as well as TE density are linked to the local recombination rate. Evidence to date suggests that TE densities correlate negatively with recombination rate, but the strength of this correlation varies across TE types [20]. At the same time, the currently most plausible explanation of the AT/GC heterogeneity in avian and mammalian genomes is a non-adaptive process called GC-biased gene conversion (gBGC), whereby increased GC% is tightly related to an increased recombination rate (recently extensively reviewed by ref. [19]). In mammals and some other vertebrates (but not birds), at least a part of the regional variation in the location of recombination hotspots can be ascribed to the activity of the protein PRDM9 [21].

One may expect that TEs contribute to the length and GC% of noncoding sequences, and continue to do so even long after they are no longer recognizable as TEs. While TE insertions are a major factor in the expansion or turnover of noncoding regions (both introns and intergenic sequences [17, 22]), the potential influence of the GC_TE_ on the host regional GC_G_ has only been comprehensively assessed for the human genome. Around 42% of the human genome is made up of retrotransposons, whereas DNA transposons only account for about 2–3%, and the insertion or accumulation of TEs depends on the isochore region involved [23]. For instance, *Alu* (the most abundant TE in human) and L1 insertions contribute to the AT/GC heterogeneity of the human genome due to their differential accumulation: *Alu* SINEs (approx. 50% GC_TE_ in their consensus sequence) reside preferentially in GC-rich regions, whereas L1 LINEs (approx. 37% GC_TE_ in their consensus sequence) reside preferentially in GC-poor regions [24]. Recognizable *Alu* elements make up 20% of GC-rich regions and 7% of GC-poor regions, whereas recognizable L1 elements make up 5% of GC-rich regions and 20% of GC-poor regions [25]. For fish, a single study briefly investigated the potential correlation between TEs and GC% along *T. nigroviridis* and *D. rerio* genomes [26]. However, they did not observe any effect of TEs on GC_G_ in *T. nigroviridis* and *D. rerio*. Three studies investigated in detail some unusual examples of GC-rich TEs in crabs [27–29] and reported different GC% between DNA transposons of marine and continental species. A bit more is known from plants and their TEs, e.g., Pack-MULEs elements in grasses specifically acquire and amplify GC-rich gene fragments [30].

In this study, we aim to bring a novel viewpoint on the vertebrate nucleotide compositional evolution by analysing the GC_TE_ of fish TEs and assessing their potential contribution to the GC_G_ and the overall nucleotide compositional landscape of their host genomes.

## Results

### Genome size positively correlates with the genomic density of TEs in fish

**Fig. 1:**
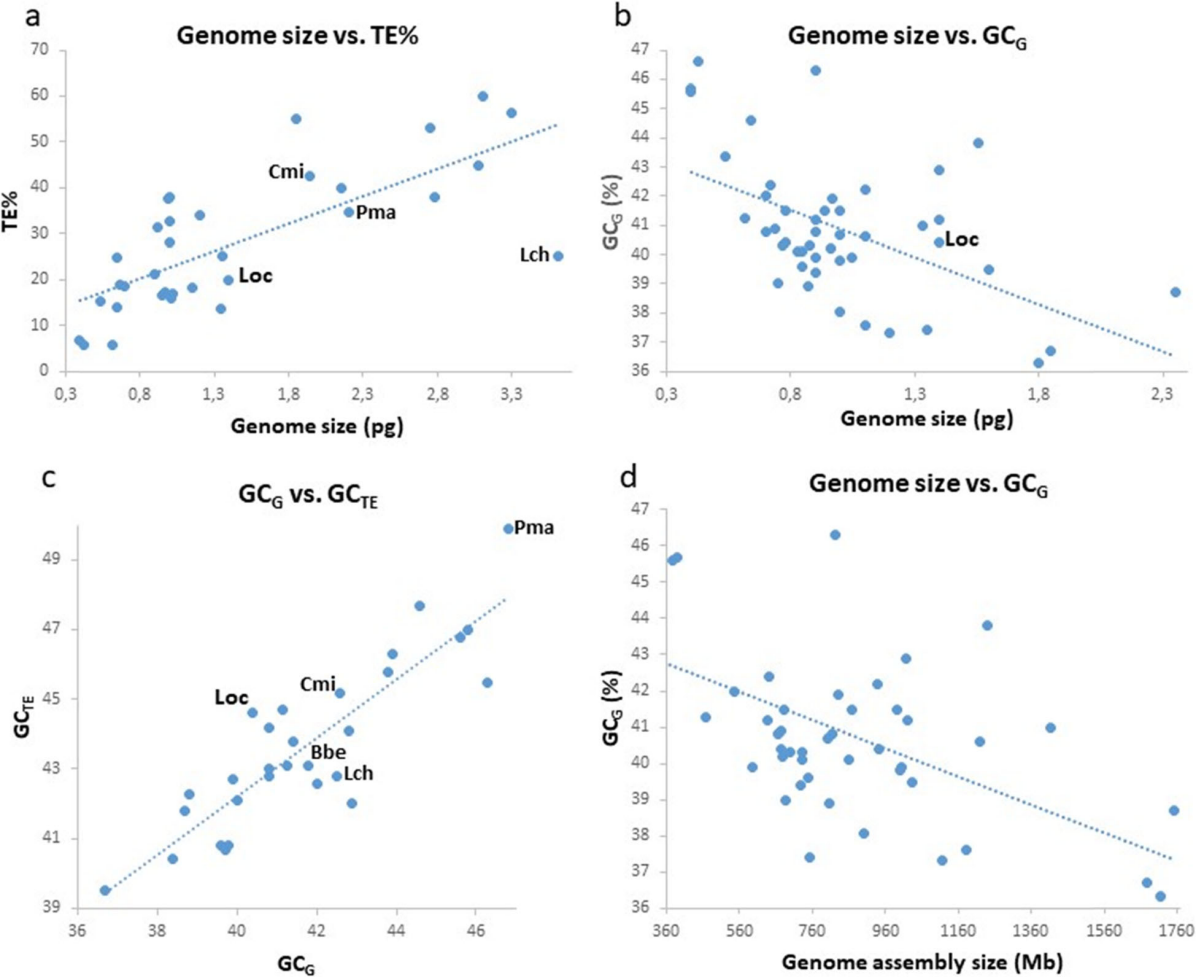
Genome size, transposable elements, and nucleotide composition. **a** Abundance of transposable elements in 29 teleosts, one non-teleost ray-finned fish (spotted gar, *L. oculatus*; Loc) with a AT/GC heterogeneous genome, one lobe-finned fish (*L. chalumnae*; Lch), one lamprey (*P. marinus*; Pma) and one shark (*C. milii*; Cmi) species related to their host genome size (genome size as C-value in picograms, pg), data from [3]. **b** GC percentage (GC%) of 46 fish genomes with available genome assemblies (excluding salmonids with their rediploidized genomes exceptionally enriched in extremely GC-rich rRNA genes [31]) negatively correlates with fish genome size based on averaged cytological measurements (C-value in pg, multiple C-value records were averaged). C-value data from the Animal Genome Size Database [32], GC% data from GenBank [33]. **c** GC% of TE consensus sequences (not accounting for their copy number within genomes) positively correlates with the overall GC% of the host genome in 25 ray-finned fish species, one lancelet (*Branchiostoma belcheri*; Bbe), one lamprey (Pma), one shark (Cmi) and one coelacanth included in FishTEDB [34]. Genomic GC% data are from GenBank [33], GC% of TEs was calculated from species-specific TE consensus sequence libraries from FishTEDB [34]. **d** GC% of genome assemblies (in Mb) of 58 fish species listed GenBank [33]

To summarize the previously reported positive correlation between fish genome size and genomic abundance of TEs [3–5, 7, 15], we generated an example plot using cytological genome size estimates, i.e. C-value in picograms (pg; Fig. 1a). Species included are 29 teleosts that underwent the teleost-specific whole-genome duplication (WGD) of which five salmonid species underwent another round of WGD, the salmonid-specific one [35]. Further, we included the spotted gar (*Lepisosteus oculatus*), i.e., a deep-branching non-teleost ray-finned fish that has not undergone any further WGD after the two basal vertebrate ones but that shows the mammalian-like situation of AT/GC heterogeneity [36]. Finally, we analysed one lamprey species (*Petromyzon marinus*), one shark (*Callorhinchus milii*) and one coelacanth (*Latimeria chalumnae*). This correlation represents an important starting point for our following considerations. Detailed lists of species analysed are in Additional files 1 and 2: Tables S1 and S2.

### Genome size negatively correlates with the genomic GC% in fish excluding salmonids

Data on GC_G_ of genome assemblies currently available in NCBI GenBank [33] and in the literature permit us to identify another crucial association – a negative correlation between fish genome size (as C-value in picograms from the Animal Genome Size Database [32]) and their genomic GC% (Fig. 1b).

To avoid any potential bias conditioned by incompleteness of currently available genome assemblies (e.g., differences in amounts of heterochromatic repeats assembled and in assembly quality sensu [37]), we compared two types of genome size datasets: one based on C-values, i.e., the non-genomics (cytological) genome size estimation (Fig. 1b) and another based on genome assembly size (Fig. 1d). Despite slight differences between these datasets, both show comparable trends, suggesting that both are usable for further analyses.

In this analysis, we excluded the eight sampled salmonid species (details in Additional file 1: Table S1) because their large genomes exhibit a salmonid-specific WGD and extremely amplified ribosomal (rRNA) genes that are exceptionally GC-rich. This feature is well known from cytogenetics [31]. Including these large and GC-enrich salmonid genomes distorts the clear correlation between GC_G_ and genome size in other teleost fish (cf. Additional file 3: Figure S1).

### GC% of TEs positively correlates with genomic GC% in fish

**Fig. 2:**
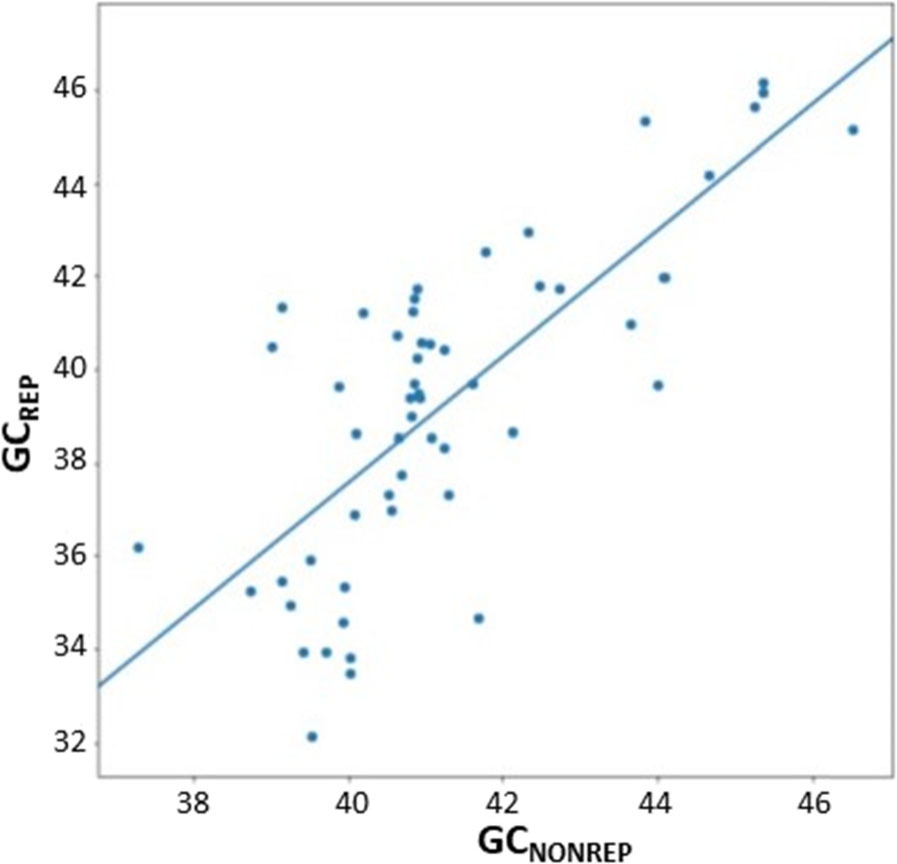
Comparison of GC% of repetitive and non-repetitive genomic fractions in 54 fish species from the Ensembl database (Release 98). The Y-axis shows GC_REP_, i.e. GC% of repeats (including low-complexity regions) masked with the RepeatMasker tool, while the X-axis shows GC_NONREP_ of the non-repetitive fraction of each assembly. Data used for this analysis are available in the Additional file 2: Table S2

Comparison of GC_TE_ with the respective GC_G_ uncovered a positive correlation. Firstly, we calculated the GC_TE_ out of the sum of individual consensus sequences of TEs annotated for each fish species from FishTEDB [34] (Fig. 1c) and not out of the entire mobilome reflecting the TEs’ copy numbers in the respective genome. As consensus sequences are approximations of the TE copies at their time point of insertion, we consider their consensus GC_TE_ to be more appropriate here because it should not reflect the genomic location of individual TE copies. Note that FishTEDB does not include any salmonid species. For comparison, we calculated GC_REP_ of repeats including low-complexity regions and compared it with the remaining non-repetitive fraction of the relevant genomes, i.e. GC_NONREP_ (Fig. 2). For this analysis, we used masked genome assemblies from the Ensembl (Release 98, [38]) as the FishTEDB lists only consensus sequences of TEs per fish species.

The GC_TE_ is mostly higher than the overall GC_G_, with two exceptions. These exceptions are cod and European eel, however, the difference is within the range of 1%, i.e., for the eel GC_G_ = 42.9% vs. GC_TE_ = 42.0% and for the cod GC_G_ = 46.3% vs. GC_TE_ = 45.5% (more details in Additional file 4: Figure S2).

### GC% varies widely among particular groups of TEs in fish

**Fig. 3:**
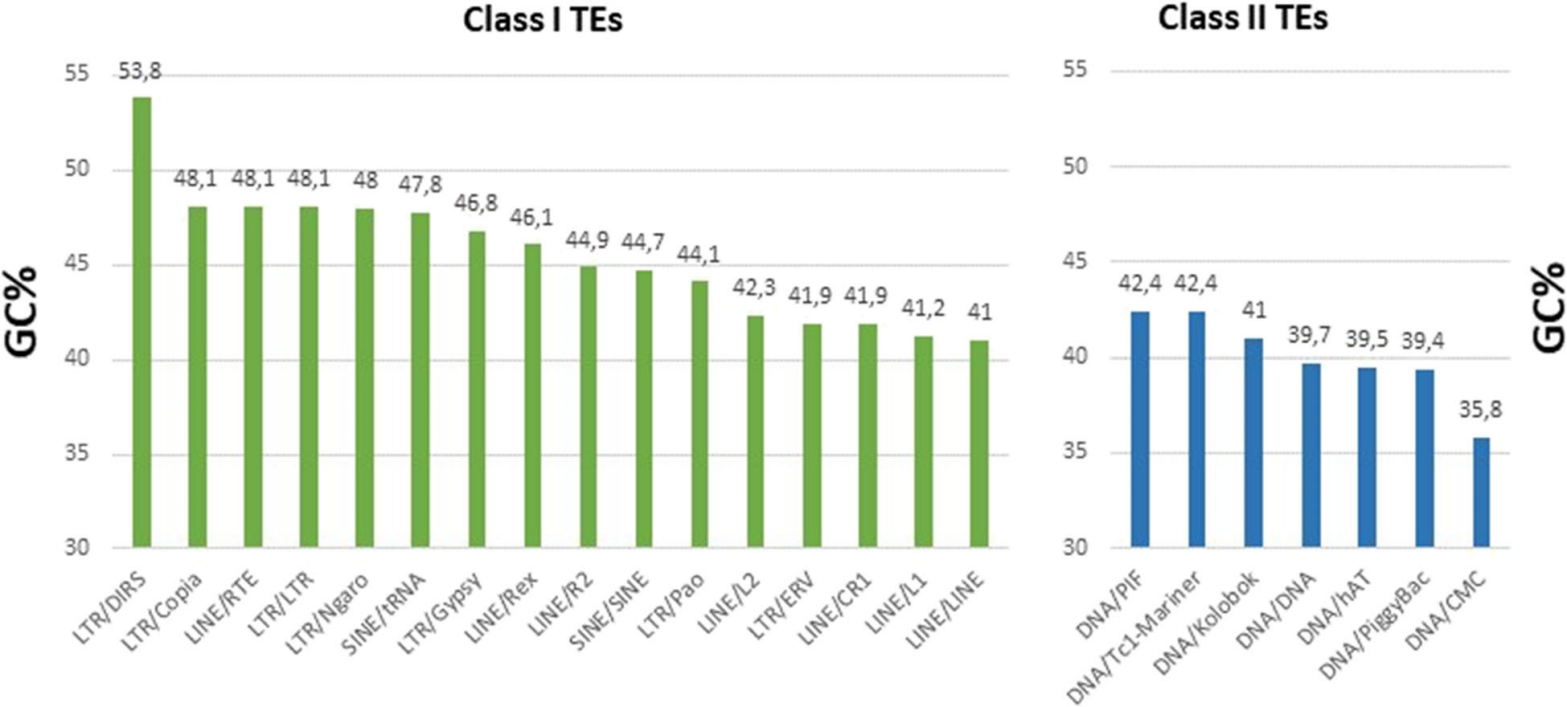
GC_TE_ in the major groups of Class I and Class II TEs, calculated as sum of GC% for all 28 fish species available in the FishTEDB database. TE consensus sequences for these calculations are from the “Browse” section of the FishTEDB database [34]

Dissecting the GC anatomy of the sum of individual TE consensus sequences in fish genomes, we further disentangled GC_TE_ of the major TE groups: Class I retrotransposons are GC-richer with an averaged consensus GC_TE_ of 45.6% than Class II DNA transposons with an averaged consensus GC_TE_ of 40.1% (Fig. 3). Within Class I, LTR retrotransposons are GC-richer than LINEs. The Class I DIRS retrotransposons are the GC-richest fish TEs with GC_TE_ of 53.8%. The Class II CMC transposons are the AT-richest fish TEs with GC_TE_ of 35.8%.

Details on the variability of species-specific GC_TE_ in 19 selected species from FishTEDB are presented in Figure S3 (Additional file 5; 16 ray-finned species, one lancelet, one shark, and one lamprey species; some species displayed in FishTEDB do not contain sequences).

### GC% of Class II DNA transposons varies heavily among different fish species

**Fig. 4:**
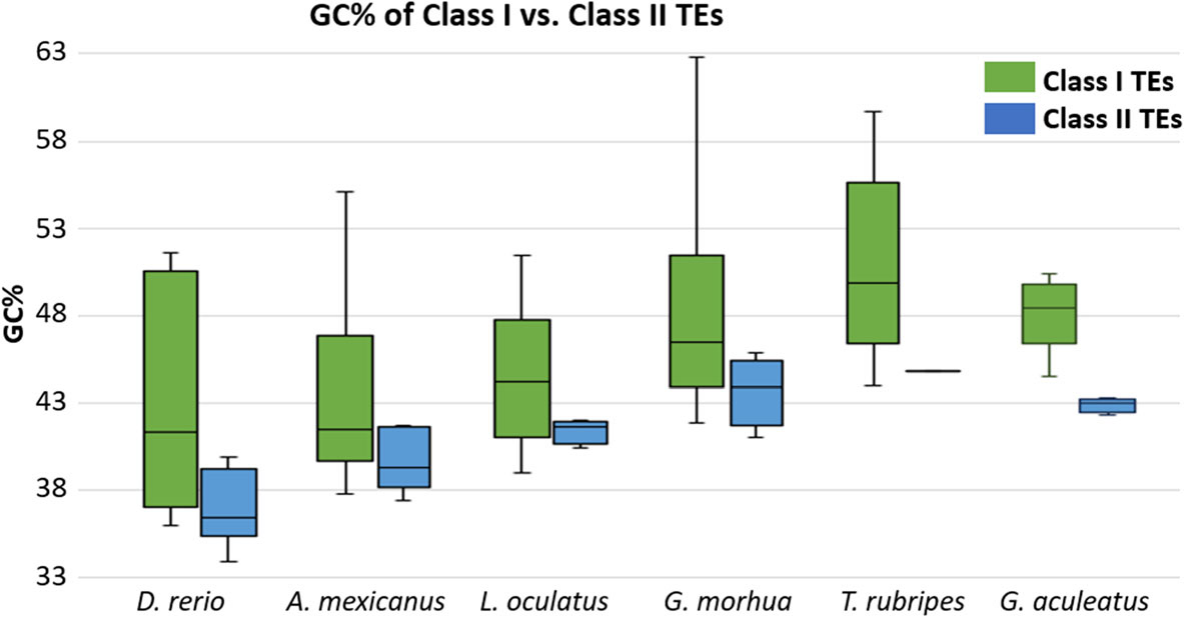
Comparison GC% between TE consensus sequences from Class I (retrotransposons) and Class II (DNA transposons) in six selected fish species (highlighted in the main text) listed in the FishTEDB database [34]

The observed variation in GC_TE_ among the major TE groups listed in the FishTEDB is particularly relevant considering that fish genomes are greatly enriched in Class II DNA transposons in contrast to avian and mammalian genomes. Therefore, we calculated the GC_TE_ of all consensus sequences of DNA transposons for 17 fish species. These data provide first insights into the GC_TE_ of fish transposons. Firstly, the compact genomes of not only pufferfishes *T. flavidus* and *T. nigroviridis* but also of cod (*G. morhua*) and stickleback (*Gasterosteus aculeatus*) show GC enrichment of their TEs as well as overall GC-richer Class II DNA transposons (Fig. 4). The same is apparent also in the non-teleost spotted gar (*L. oculatus*) with its AT/GC heterogeneous genome and an unusually high GC_TE_ in comparison with teleosts. The opposite situation occurs in teleosts with larger genomes such as *D. rerio* and *Astyanax mexicanus*: DNA transposons are GC-poor(er) as well as the overall GC_G_ and GC_TE_ are lower.

## Discussion

Recent studies on the relative contribution of TEs to genome size in fish [3, 4, 7, 39] have become an important starting point for us to understand the evolution of nucleotide composition. The above listed results raise crucial questions about the contribution of the mobilome GC% to the entire genomic GC% and to the nucleotide compositional landscape. This has been so far addressed only for the human genome [22]. Here, we show that utilizing purely genomic data for approximating genome size (assembly vs. C-value) and GC% yield reproducible and comparable data suitable for assessing nucleotide composition of host genomes and their respective TEs. The ever-increasing number of available assemblies and TE annotations for fish and other vertebrates has now become sufficient to begin to address the questions raised here.

### GC richness vs. AT/GC heterogeneity and TEs

It is necessary to distinguish between an overall genomic GC-richness, i.e., GC_G_, and the avian or mammalian situation of AT/GC heterogeneity (recorded also in non-teleost gars [36]). This entails an alternation of GC-rich and GC-poor regions along linkage groups, thus forming banding patterns on chromosomes upon an AT/GC-specific staining (recently reviewed by [36]). In the case of AT/GC heterogeneity, the overall GC_G_ can be even lower than is in cases of AT/GC homogeneity typical for fish genomes as shown below. Considering that all of the currently available vertebrate genome assemblies contain gaps due to either repeat-rich or GC-rich regions [37], fish with GC-rich genomes might actually be even GC-richer than currently estimated, and potentially even more GC-rich than mammalian and avian genomes. This is indicated by the following examples: the human (GC_G_ = 40.9%), mouse (GC_G_ = 42.5%), and even chicken (GC_G_ = 41.9%) genomes are GC-poorer than cod (GC_G_ = 46.3%) and three pufferfish species (GC_G_ = 45.6, 45.7% and GC_G_ = 46.6% respectively). However, note the situation in the non-teleost spotted gar with GC_G_ = 40.4% and AT/GC heterogeneity. The total length of its available assembly is merely 945.878 Mb [33], which is remarkably incomplete in comparison with the cytological genome size estimate of 1.4 pg [32]. Nevertheless, the AT/GC heterogeneity evidenced cytogenetically was also confirmed using genomic data [36].

The smaller and GC-rich(er) fish genomes also contain lower TE densities (or lower densities of GC-poor TEs) and/or GC-rich (er) TEs. The fact that the averaged GC% of consensus sequences from all TE families is generally higher than the entire genomic GC% suggests that TE spread and accumulation might contribute to the overall GC_G_ in fish. This is further supported by our observation that genomes with a higher GC% of the repetitive genomic fraction (i.e., TEs and other repeats; GC_REP_) have a higher GC_NONREP_, i.e., GC% of the non-repetitive rest of the genome. However, due to the broad range of GC_TE_ of major groups of TEs in different species (Fig. 3), the activity and abundance of GC-poor(er) DNA transposons might also contribute to the AT/GC homogeneity in fish, assuming they accumulated more homogenously, compared to the AT/GC heterogeneity in avian and mammalian genomes that usually lack activity of DNA transposons.

### How could TEs shape the host nucleotide compositional landscape?

Considering our findings, we anticipate at least three possible ways how TEs could influence the host nucleotide compositional landscape: 1) TEs shape it through inserting their “own” GC in a new context (i.e., increasing GC% of the region if they have high GC; lowering GC% of the region of they have low GC); 2) TEs shape nearby GC% through “spillover” of CpG methylation (‘sloping shores’ model of [40]), leading to CpG hypermutation and thus decrease of nearby GC%; and 3) some TEs might contain sequence motifs that increase or decrease the local recombination landscape and thus the strength of GC-biased gene conversion. There are however many more questions about GC% of TEs to be answered: Are quantitatively larger mobilomes as GC-poor as larger host genomes are overall? Why are DNA transposons GC-poor? Why are some DNA transposons GC-poorer than others and only so in some species?

### Conclusion and perspectives

Here we have shown that nucleotide composition of TEs and their interplay with host genomes is an unexplored part of genome biology. The GC-poor DNA transposons predominant in fish genomes and nearly absent in avian and mammalian genomes might have indeed contributed to shaping the nucleotide compositional landscape in vertebrates. Only the GC-heterogeneous gar and the GC-enriched pufferfishes possess GC-richer TEs and fewer DNA transposons. At the same time, among others the GC-poor genome of zebrafish possesses the GC-poorest TEs. Hence, it is possible that DNA transposon spreading and accumulation has actively contributed to the overall GC homogenization of fish genomes. On the other hand, replacement of DNA transposons by retrotransposons in avian and mammalian genomes might have contributed to their AT/GC heterogeneity through differential accumulation across chromosomes. The GC content of TEs should thus be considered as one of the factors potentially shaping the nucleotide compositional landscape in vertebrates and requires further investigations in detail. The next step envisaged is a qualitative analysis of the contribution GC% of individual TE insertions to the GC% of host genomes while accounting for TE copy number. This step can be combined with cytogenetic data to investigate the chromosomal distribution of various TEs and their potential contribution to the GC homogenization of fish genomes. With 55 fish species genome assemblies recently introduced by the 98th release of Ensembl (November 2019 [38]) and numerous others, such comprehensive analyses now appear feasible.

## Methods

All species analysed in datasets produced for this study are listed in the Additional file 1: Table S1 and the datasets supporting the conclusions of this article are included in the Additional file 2: Table S2. We obtained genome size data as C-values from the www.genomesize.com database [32]. At this stage, diverse sources of datasets and databases (ref. [3], Animal Genome Size Database [32], GenBank [33], FishTEDB [34]) list different sets of fish species of which only some have been analysed for TEs. Assembly size data in Mb were obtained from the NCBI GenBank records of sequenced genomes [33]. Proportions of TEs in fish genomes were obtained from ref. [3] and compared with ref. [7]. Sequences of annotated fish TEs were obtained from Fish TE database http://www.fishtedb.org [34] and from the Repbase database at www.girinst.org [41]. Further data were extracted from literature as listed in the Additional file 2: Table S2. We used custom Python scripts to extract GC_REP_ (repeats including low-complexity regions) of fish genomes in the Ensembl database (https://www.ensembl.org/ [38]) and compared to GC% of the rest of the genome assembly (GC_NONREP_), i.e. the non-repetitive fraction. The scripts are available at the GitHub repository https://github.com/bioinfohk/GC_TE/blob/master/GC_softmasked_genomesFISH.ipynb.

## Supplementary information

**Additional file 1: Table S1.** Species overview and their counts.

**Additional file 2: Table S2.** Datasets used for generating Figs. 1, 2, 3, 4 and Additional files 3 and 4: Figures S1-S2.

**Additional file 3: Figure S1.** Analysis of genome size vs. GC_G_ including salmonids (for comparison with Fig. 1b).

**Additional file 4: Figure S2.** Comparison of GC_G_ and GC_TE_ in 29 fish species (ray-finned fish and outgroups lancelet *Branchiostoma belcheri*, lamprey *Petromyzon marinus*, shark *Callorhinchus milii*, and coelacanth *Latimeria chalumnae*) listed in the FishTEDB [36]. In only two species analysed, GC_TE_ (orange) is lower than GC_G_ (blue; *A. anguilla* and *G. morhua*). Based on the dataset for Fig. 1c in Additional file 2.

**Additional file 5: Figure S3.** Species-specific comparisons of GC_TE_ between Class I and Class II TEs.

## Data Availability

All data generated or analysed during this study are included in this published article and its supplementary information files.

## Abbreviations

GC%: Percentage of G + C bases, i.e., the molar ratio of guanine and cytosine in DNA
GC_G_: GC% of the whole genome
GC_NONREP_: GC% of the non-repetitive fraction of genome assemblies in Ensembl
GC_REP_: GC% of the repetitive fraction of genome assemblies in Ensembl
GC_TE_: GC% of TE consensus sequences
GS: Genome size
LINE: Long interspersed element
LTR: Long terminal repeat
MLE: *Mariner*-like element
SINE: Short interspersed element
TE: Transposable element
WGD: Whole genome duplication
