# Supplementary figures and images for: Nucleotide composition of transposable elements likely contributes to AT/GC compositional homogeneity of teleost fish genomes
Source: Mob DNA. 2019 Dec 12;10:49. doi: 10.1186/s13100-019-0195-y (PMC6909575; doi:10.1186/s13100-019-0195-y)

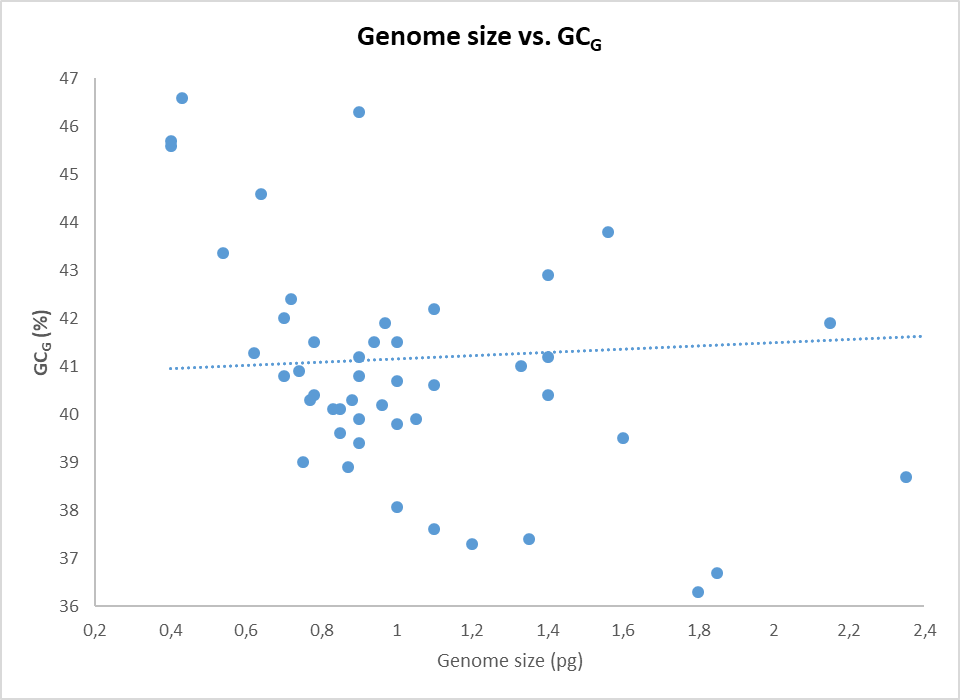

Supplement: Supplementary file 3 — Additional file 3: Figure S1. Analysis of genome size vs. GCG including salmonids (for comparison with Fig. 1b). [file 13100_2019_195_MOESM3_ESM.docx]

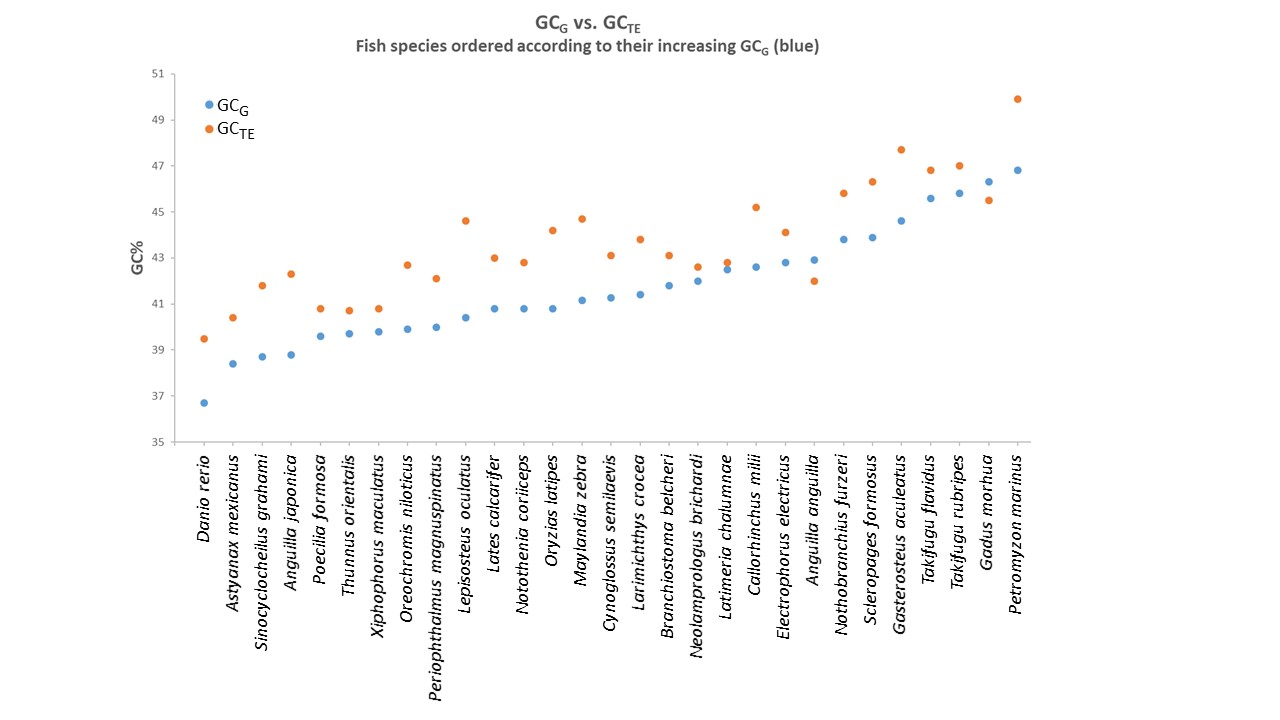

Supplement: Supplementary file 4 — Additional file 4: Figure S2. Comparison of GCG and GCTE in 29 fish species (ray-finned fish and outgroups lancelet Branchiostoma belcheri, lamprey Petromyzon marinus, shark Callorhinchus milii, and coelacanth Latimeria chalumnae) listed in the FishTEDB [36]. In only two species analysed, GCTE (orange) is lower than GCG (blue; A. anguilla and G. morhua). Based on the dataset for Fig. 1c in Additional file 2. [file 13100_2019_195_MOESM4_ESM.docx]
