# Supplementary material for: Nucleotide composition of transposable elements likely contributes to AT/GC compositional homogeneity of teleost fish genomes
Source: Mob DNA. 2019 Dec 12;10:49. doi: 10.1186/s13100-019-0195-y (PMC6909575; doi:10.1186/s13100-019-0195-y)
Supplement: Supplementary file 5 — Additional file 5: Figure S3. Species-specific comparisons of GCTE between Class I and Class II TEs. [file 13100_2019_195_MOESM5_ESM.pdf]

Additional File 5

Fig. S3 Species specific comparisons of GC_{TE} between Class I and Class II in following species:

Takifugu flavidus
Takifugu rubripes
Danio rerio
Lepisosteus oculatus
Thunnus orientalis
Xiphophorus maculatus
Cynoglossus semilaevis
Anguilla anguilla
Ctenopharyngodon idellus
Astyanax mexicanus
Gadus morhua
Gasterosteus aculeatus
Oreochromis niloticus
Oryzias latipes
Scleropages formosus
Poecilia formosa
Branchiostoma belcheri
Petromyzon marinus
Callorhinchus mili

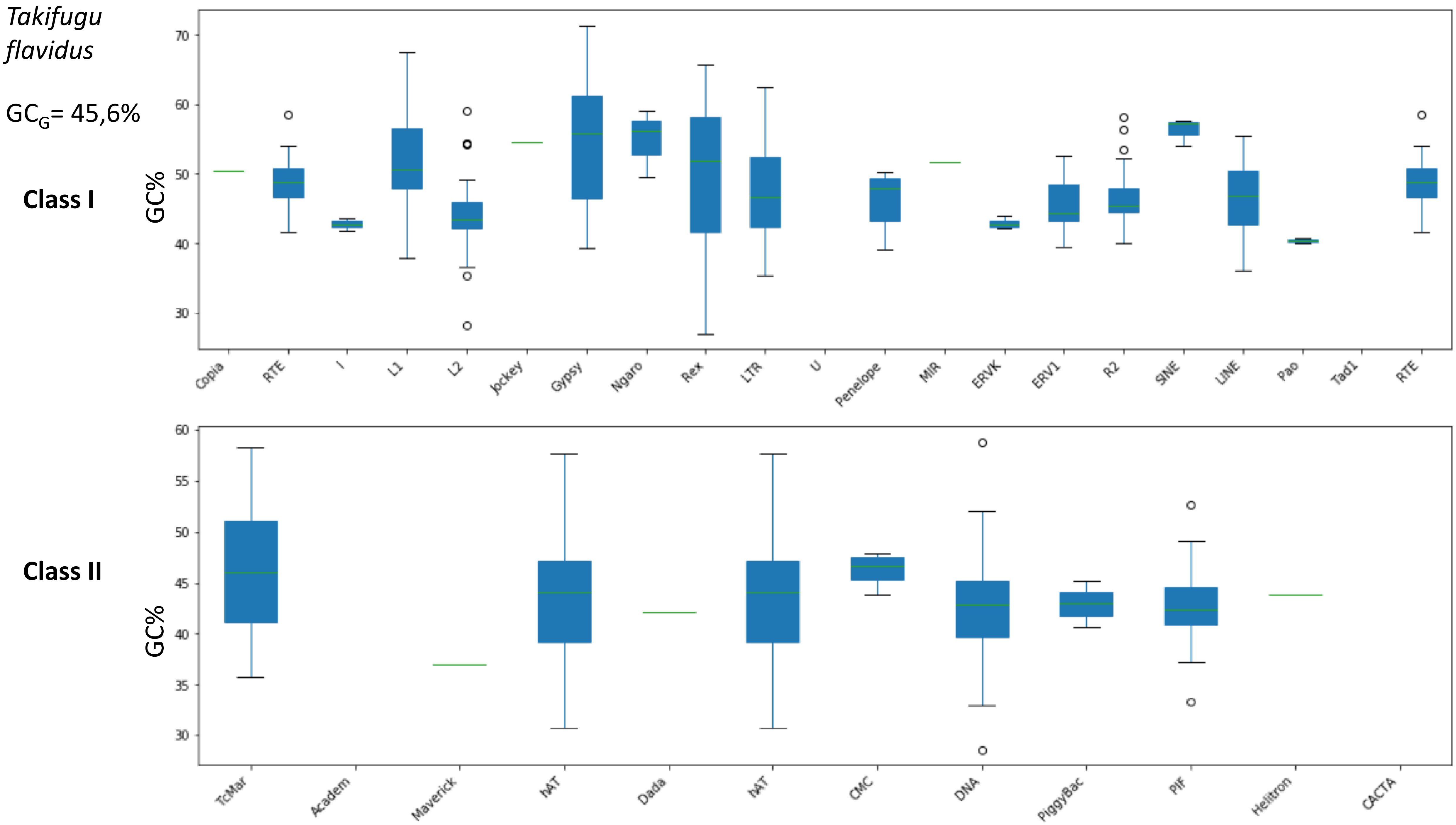

Takifugu flavidus
GCG= 45,6%
Class I
Class II
GC%
70
60
50
40
30
Copia
RTE
I
L1
L2
Jockey
Gypsy
Ngaro
Rex
LTR
U
Penelope
MIR
ERVK
ERV1
R2
SINE
LINE
Pao
Tad1
RTE
60
55
50
45
40
35
30
TcMar
Academ
Maverick
hAT
Dada
hAT
CMC
DNA
PiggyBac
PIF
Helitron
CACTA

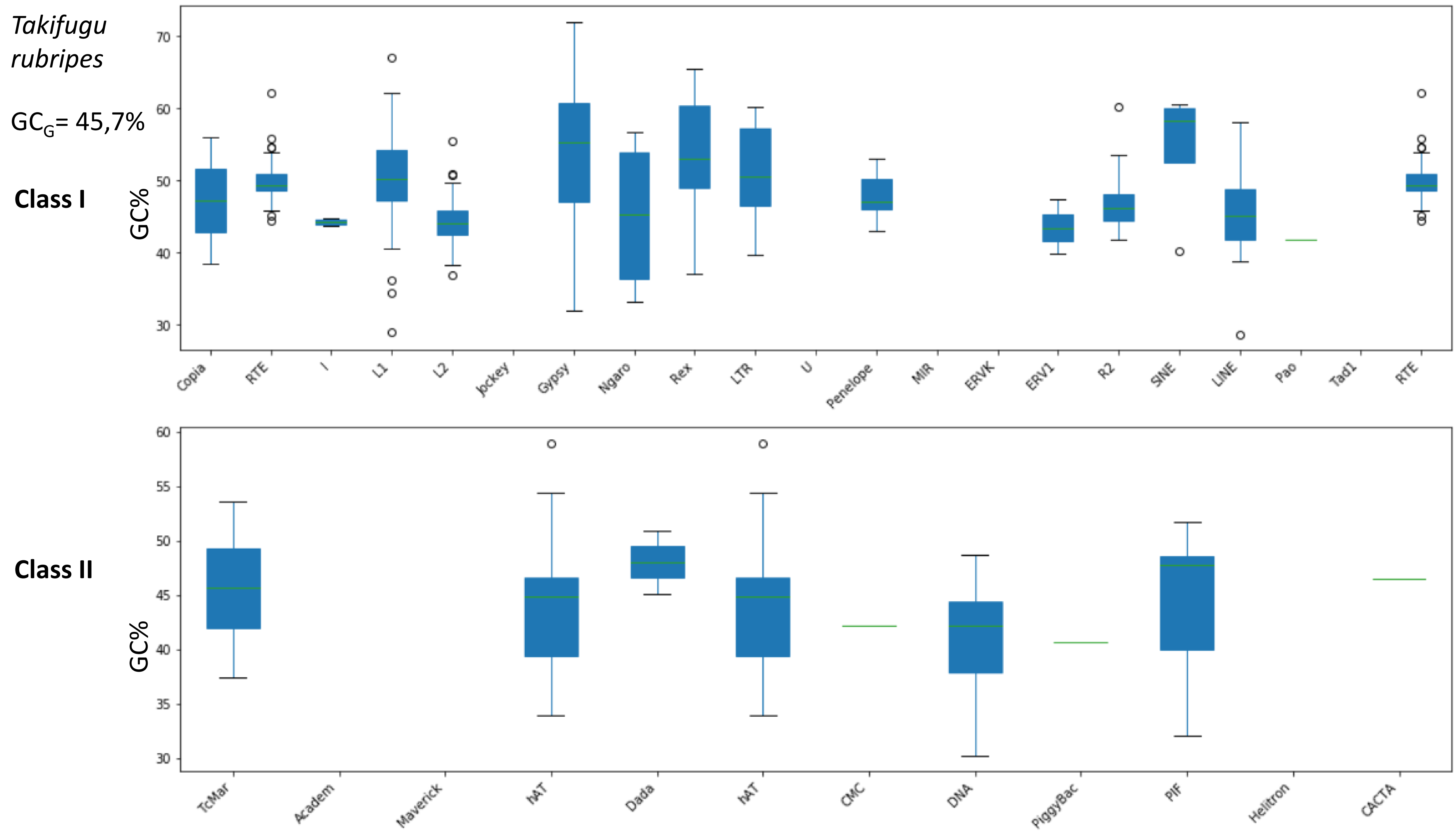

Takifugu rubripes
GC$_G$= 45,7%
Class I
GC%
70
60
50
40
30
Copia
RTE
I
L1
L2
Jockey
Gypsy
Ngaro
Rex
LTR
U
Penelope
MIR
ERVK
ERV1
R2
SINE
LINE
Pao
Tad1
RTE
Class II
GC%
60
55
50
45
40
35
30
TcMar
Academ
Maverick
hAT
Dada
hAT
CMC
DNA
PiggyBac
PIF
Helitron
CACTA

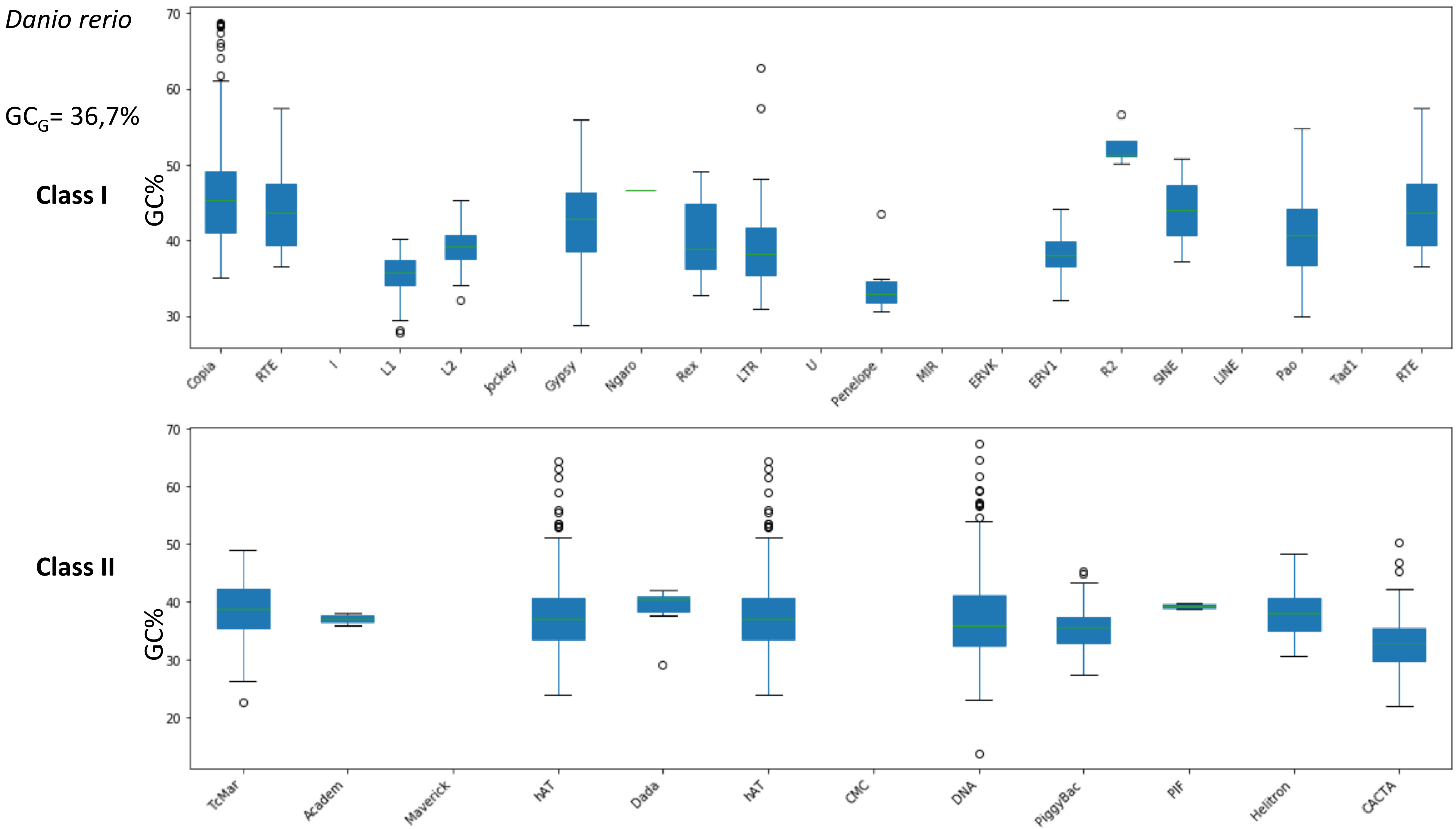

Danio rerio
GC_G= 36,7%
Class I
GC%
70
60
50
40
30
Copia
RTE
I
L1
L2
Jockey
Gypsy
Ngaro
Rex
LTR
U
Penelope
MIR
ERVK
ERV1
R2
SINE
LINE
Pao
Tad1
RTE
Class II
GC%
70
60
50
40
30
20
TcMar
Academ
Maverick
hAT
Dada
hAT
CMC
DNA
PiggyBac
PIF
Helitron
CACTA

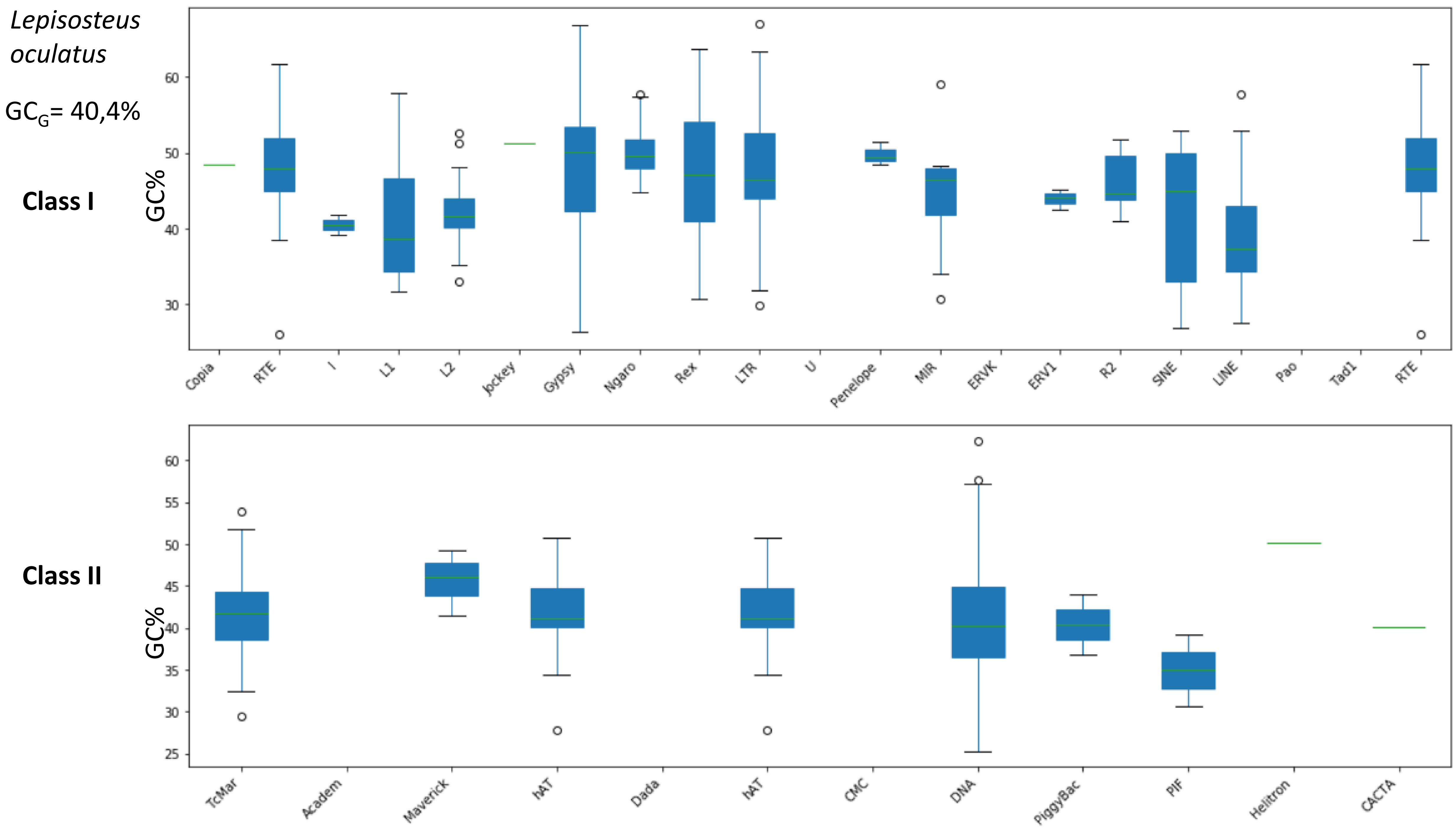

Lepisosteus oculatus
GC$_G$= 40,4%
Class I
GC%
Copia
RTE
I
L1
L2
Jockey
Gypsy
Ngaro
Rex
LTR
U
Penelope
MIR
ERVK
ERV1
R2
SINE
LINE
Pao
Tad1
RTE
Class II
GC%
TcMar
Academ
Maverick
hAT
Dada
hAT
CMC
DNA
PiggyBac
PIF
Helitron
CACTA

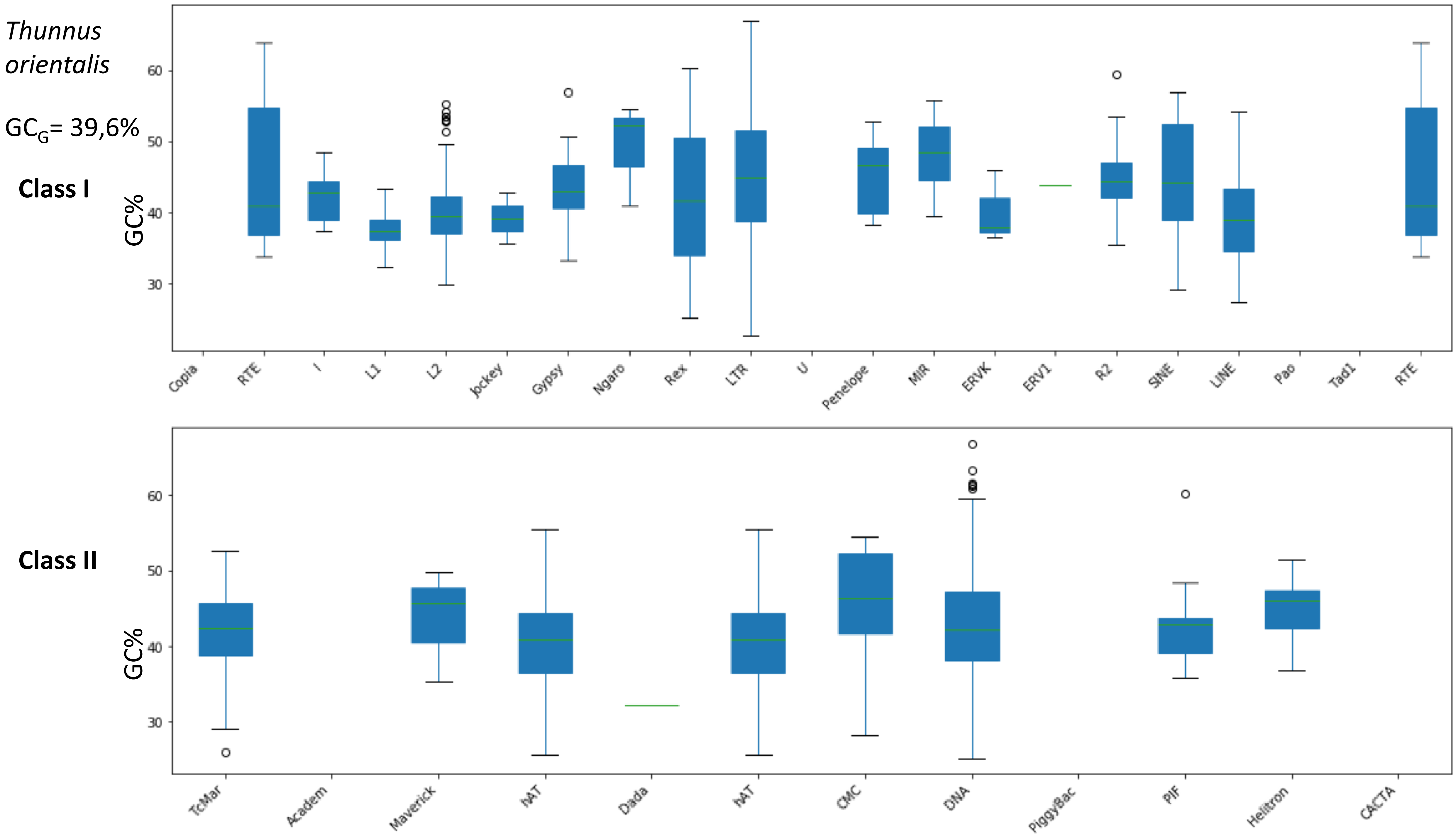

Thunnus orientalis
GCG= 39,6%
Class I
GC%
60
50
40
30
Copia
RTE
I
L1
L2
Jockey
Gypsy
Ngaro
Rex
LTR
U
Penelope
MIR
ERVK
ERV1
R2
SINE
LINE
Pao
Tad1
RTE
Class II
GC%
60
50
40
30
TcMar
Academ
Maverick
hAT
Dada
hAT
CMC
DNA
PiggyBac
PIF
Helitron
CACTA

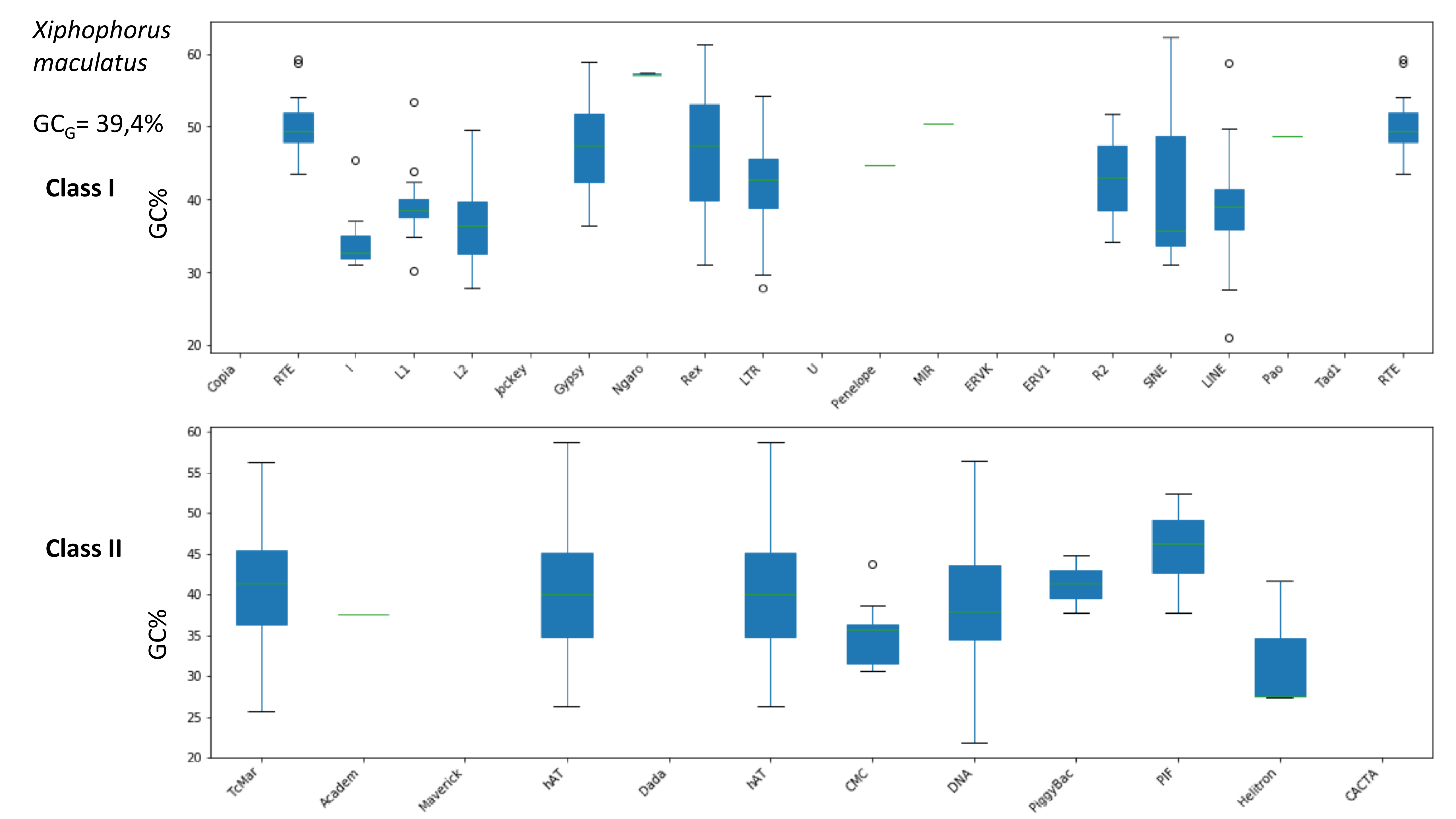

Xiphophorus maculatus
GC_G= 39,4%
Class I
Class II
GC%
GC%
60
50
40
30
20
Copia
RTE
I
L1
L2
Jockey
Gypsy
Ngaro
Rex
LTR
U
Penelope
MIR
ERVK
ERV1
R2
SINE
LINE
Pao
Tad1
RTE
60
55
50
45
40
35
30
25
20
TcMar
Academ
Maverick
hAT
Dada
hAT
CMC
DNA
PiggyBac
PIF
Helitron
CACTA

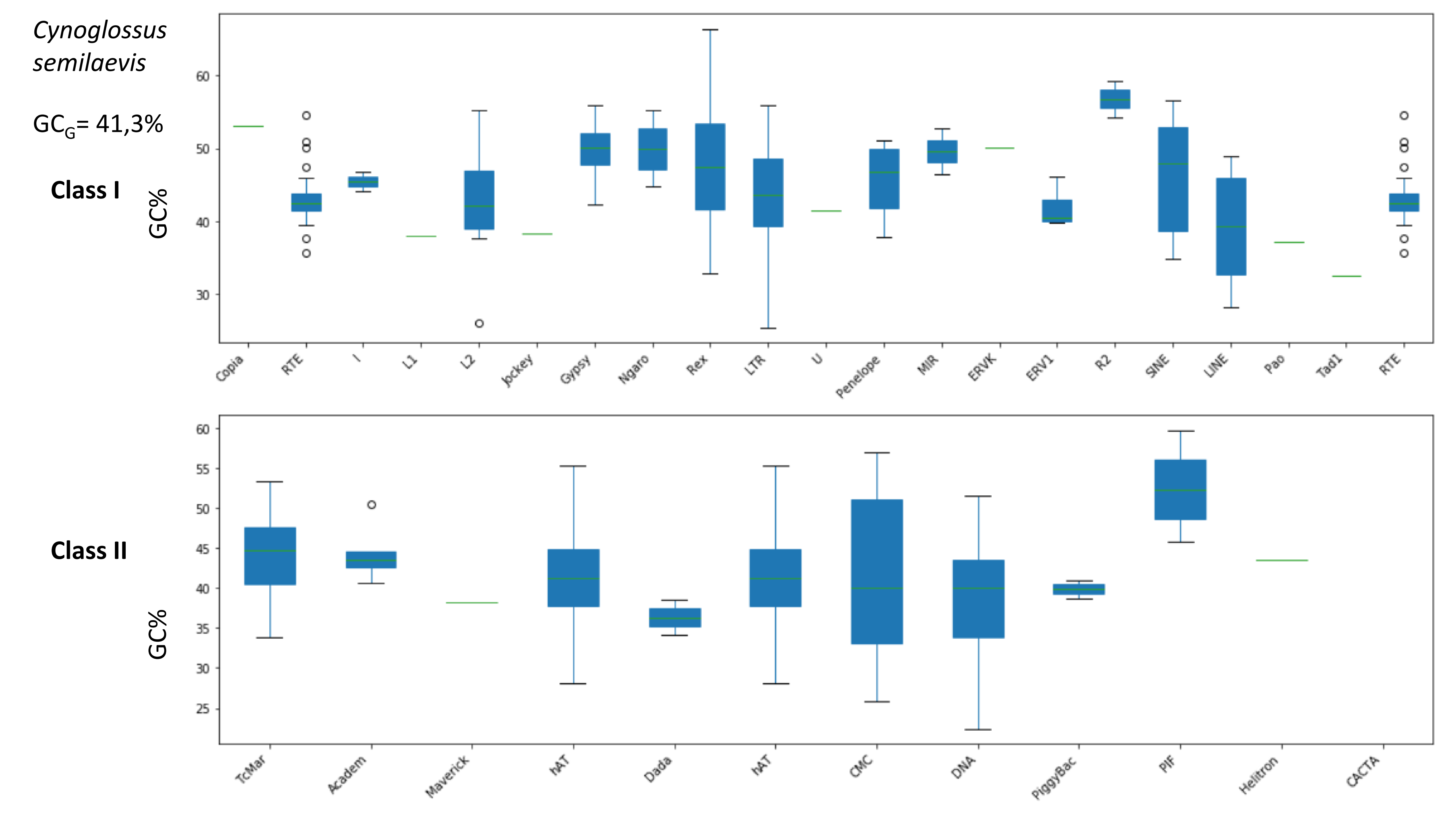

Cynoglossus semilaevis
GC_G= 41,3%
Class I
GC%
60
50
40
30
Copia
RTE
I
L1
L2
Jockey
Gypsy
Ngaro
Rex
LTR
U
Penelope
MIR
ERVK
ERV1
R2
SINE
LINE
Pao
Tad1
RTE
Class II
GC%
60
55
50
45
40
35
30
25
TcMar
Academ
Maverick
hAT
Dada
hAT
CMC
DNA
PiggyBac
PIF
Helitron
CACTA

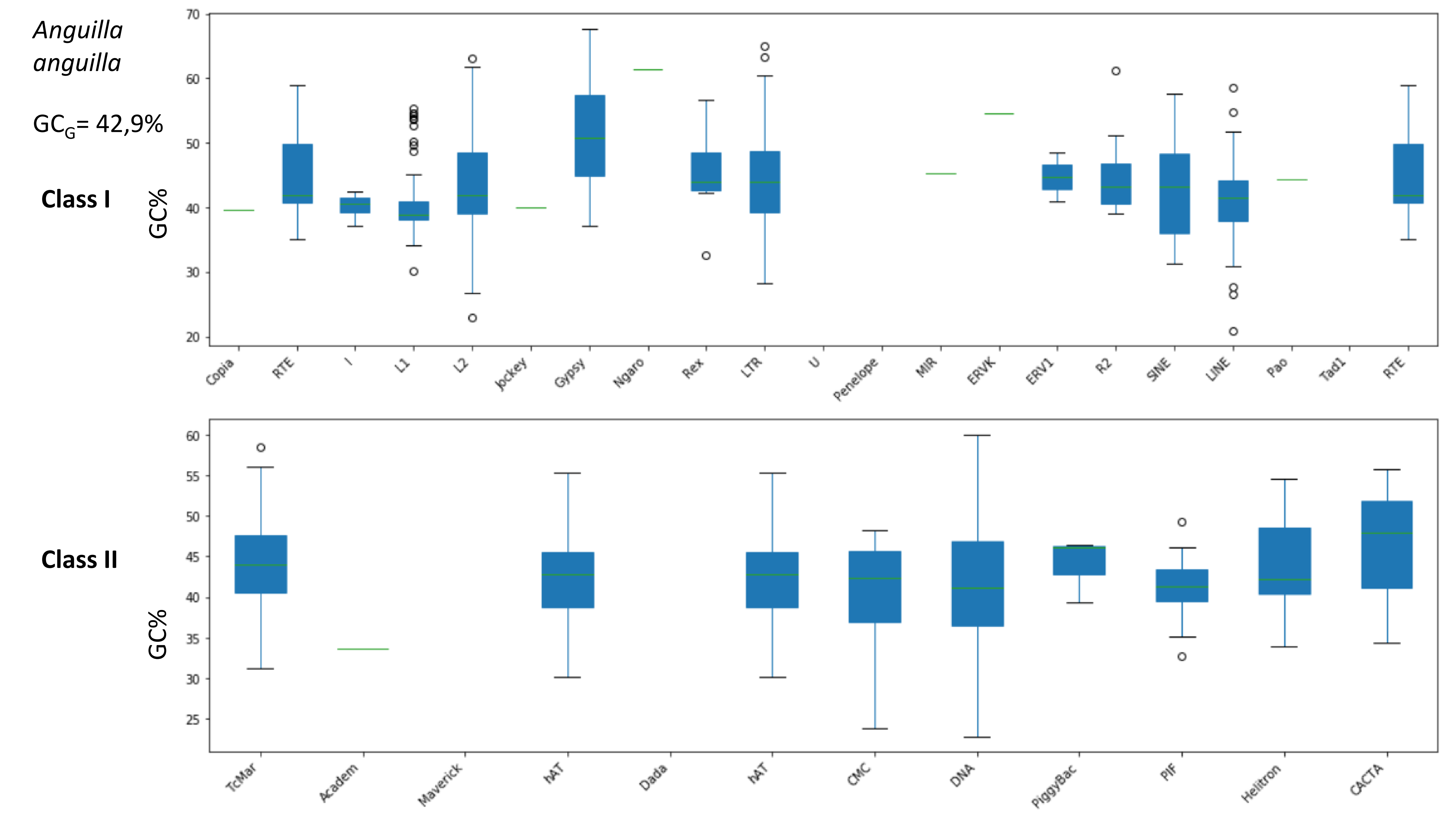

Anguilla anguilla
GC_G= 42,9%
Class I
GC%
70
60
50
40
30
20
Copia
RTE
I
L1
L2
Jockey
Gypsy
Ngaro
Rex
LTR
U
Penelope
MIR
ERVK
ERV1
R2
SINE
LINE
Pao
Tad1
RTE
Class II
GC%
60
55
50
45
40
35
30
25
TcMar
Academ
Maverick
hAT
Dada
hAT
CMC
DNA
PiggyBac
PIF
Helitron
CACTA

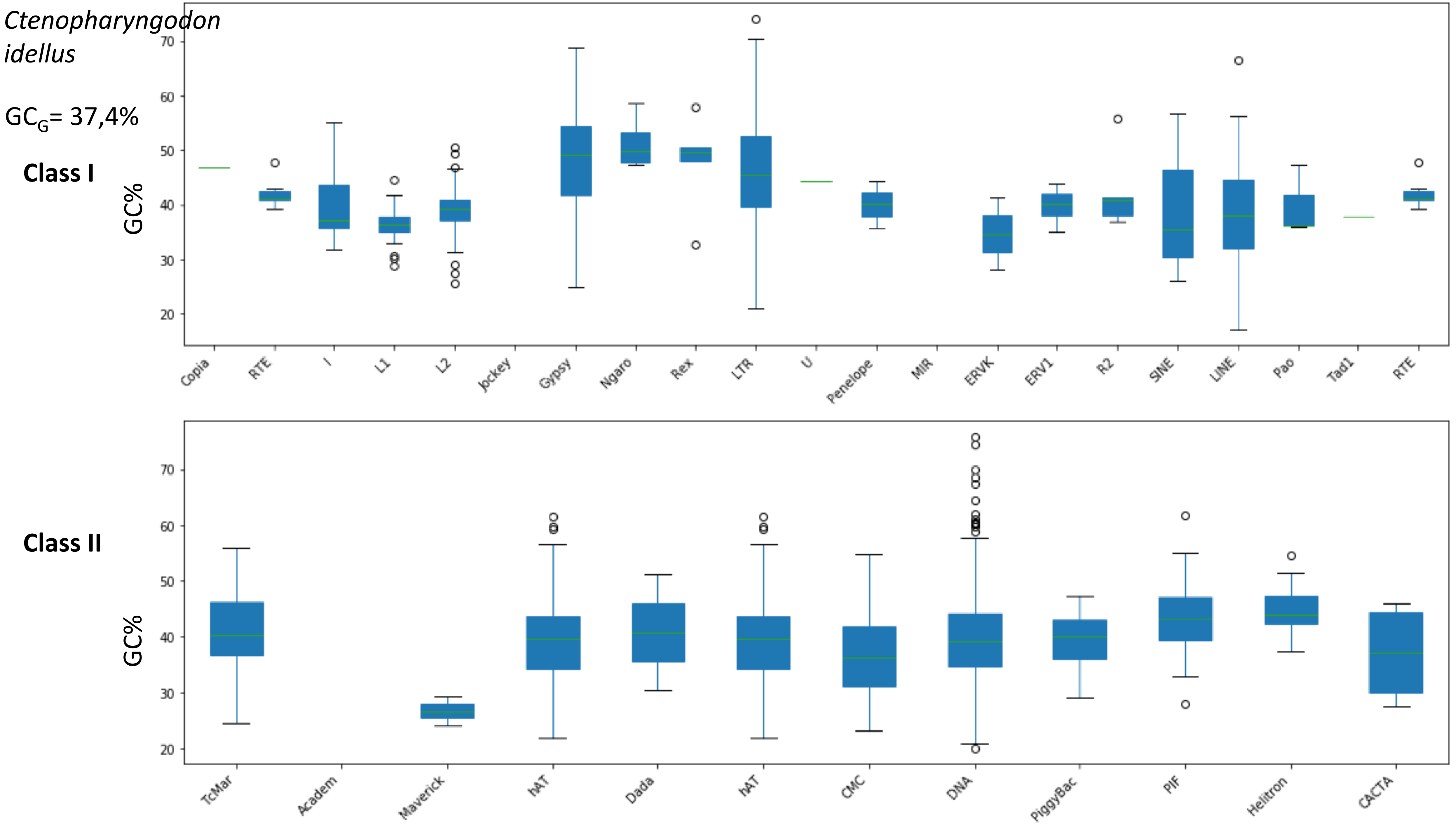

Ctenopharyngodon idellus
GCG= 37,4%
Class I
Class II
GC%
Copia
RTE
I
L1
L2
Jockey
Gypsy
Ngaro
Rex
LTR
U
Penelope
MIR
ERVK
ERV1
R2
SINE
LINE
Pao
Tad1
RTE
TcMar
Academ
Maverick
hAT
Dada
hAT
CMC
DNA
PiggyBac
PIF
Helitron
CACTA

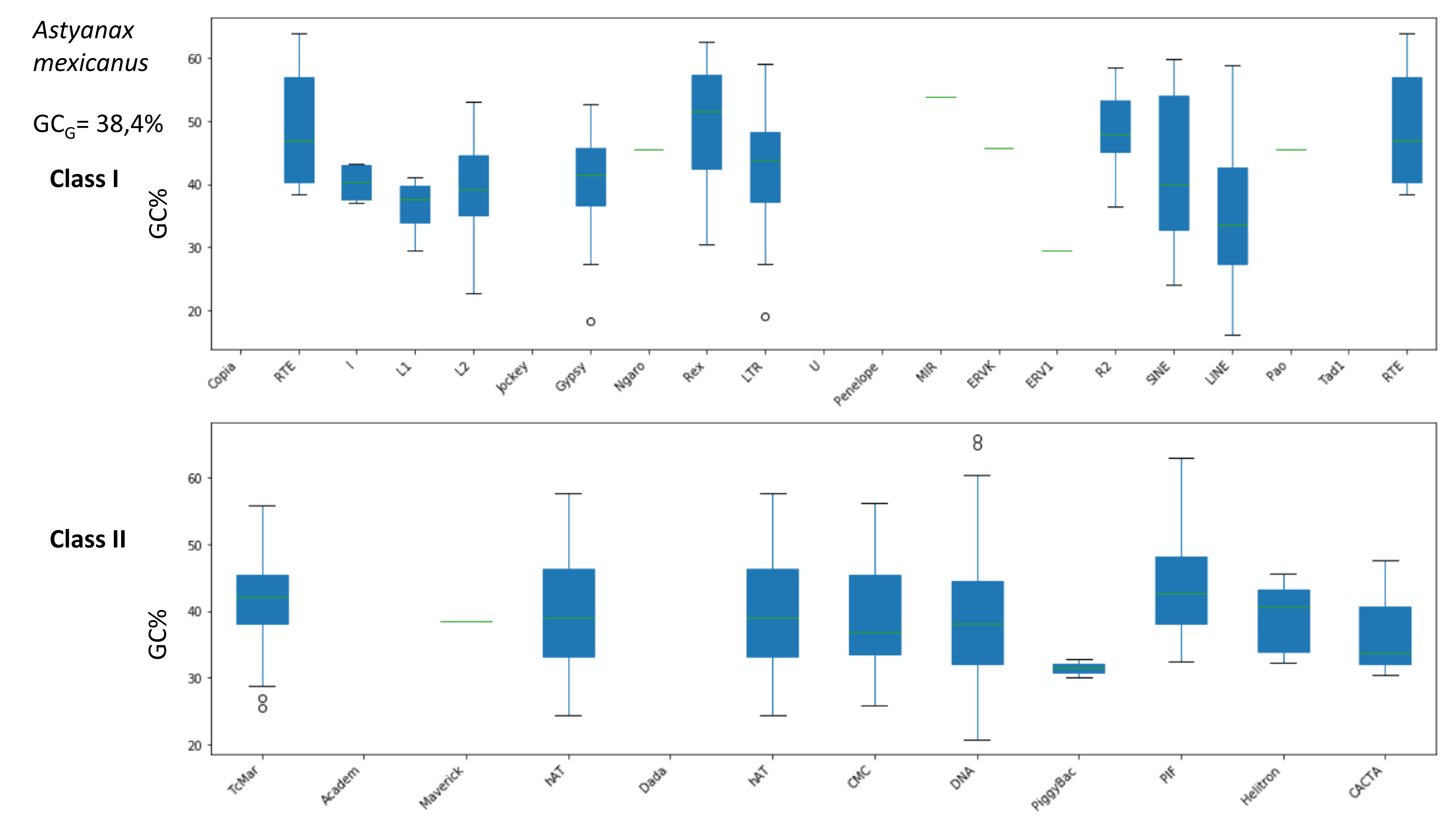

Astyanax mexicanus
GCG= 38,4%
Class I
Class II
GC%
GC%
60
50
40
30
20
Copia
RTE
I
L1
L2
Jockey
Gypsy
Ngaro
Rex
LTR
U
Penelope
MIR
ERVK
ERV1
R2
SINE
LINE
Pao
Tad1
RTE
TcMar
Academ
Maverick
hAT
Dada
hAT
CMC
DNA
PiggyBac
PIF
Helitron
CACTA

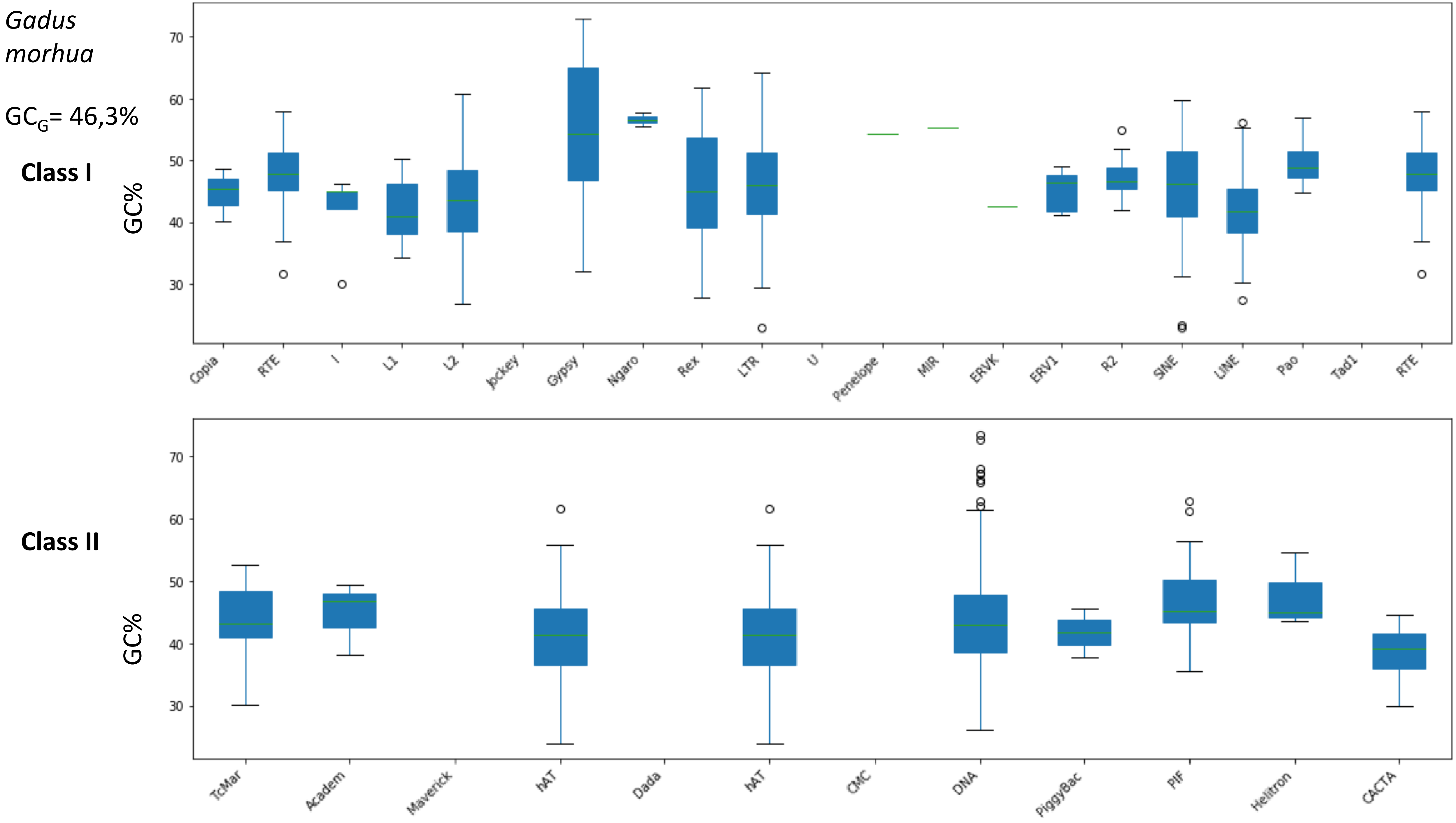
Gadus morhua
GC$_G$= 46,3%
Class I
GC%
70
60
50
40
30
Copia
RTE
I
L1
L2
Jockey
Gypsy
Ngaro
Rex
LTR
U
Penelope
MIR
ERVK
ERV1
R2
SINE
LINE
Pao
Tad1
RTE
Class II
GC%
70
60
50
40
30
TcMar
Academ
Maverick
hAT
Dada
hAT
CMC
DNA
PiggyBac
PIF
Helitron
CACTA

Gasterosteus aculeatus

GC_G= 44,6%

Class I

Class II

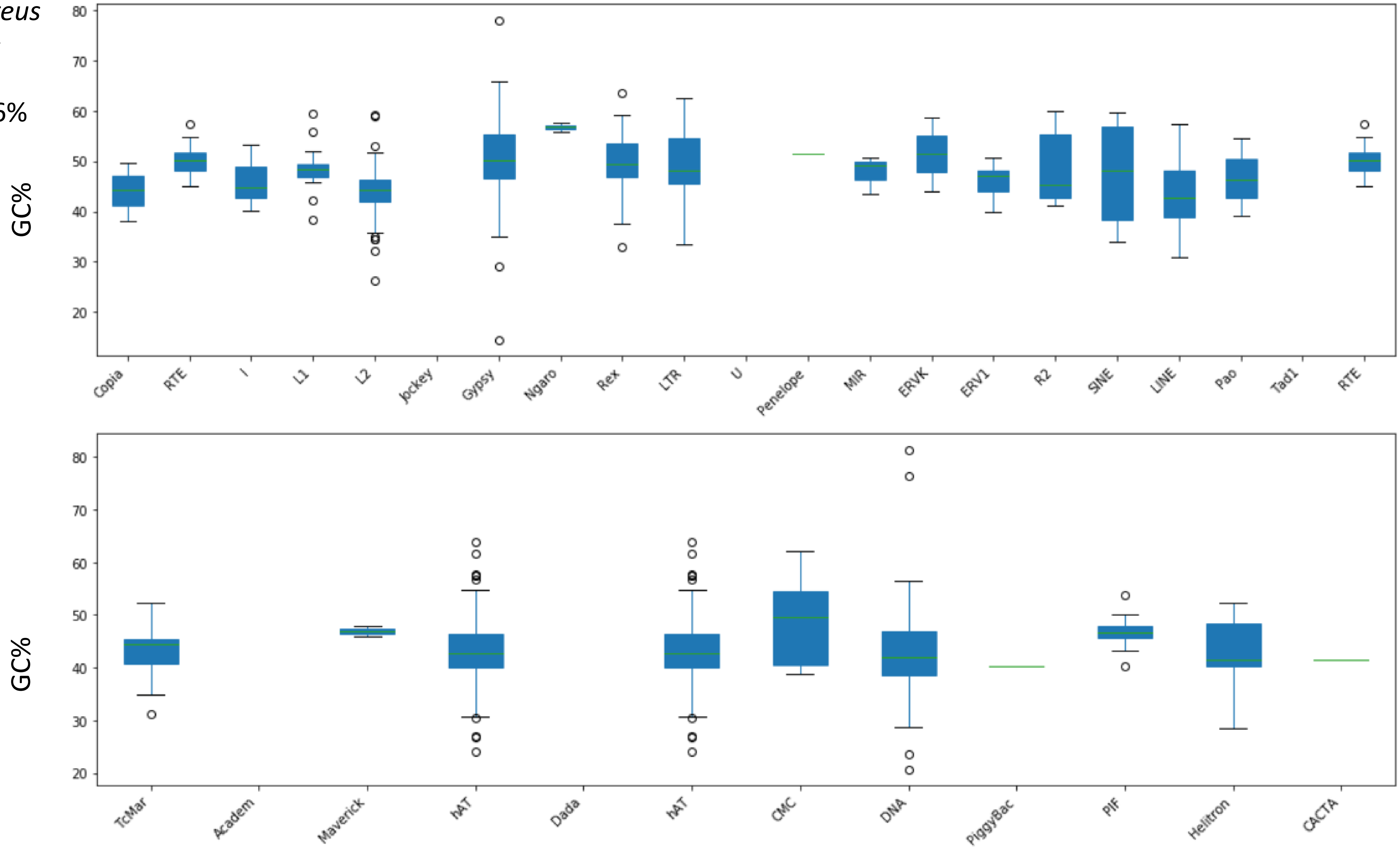

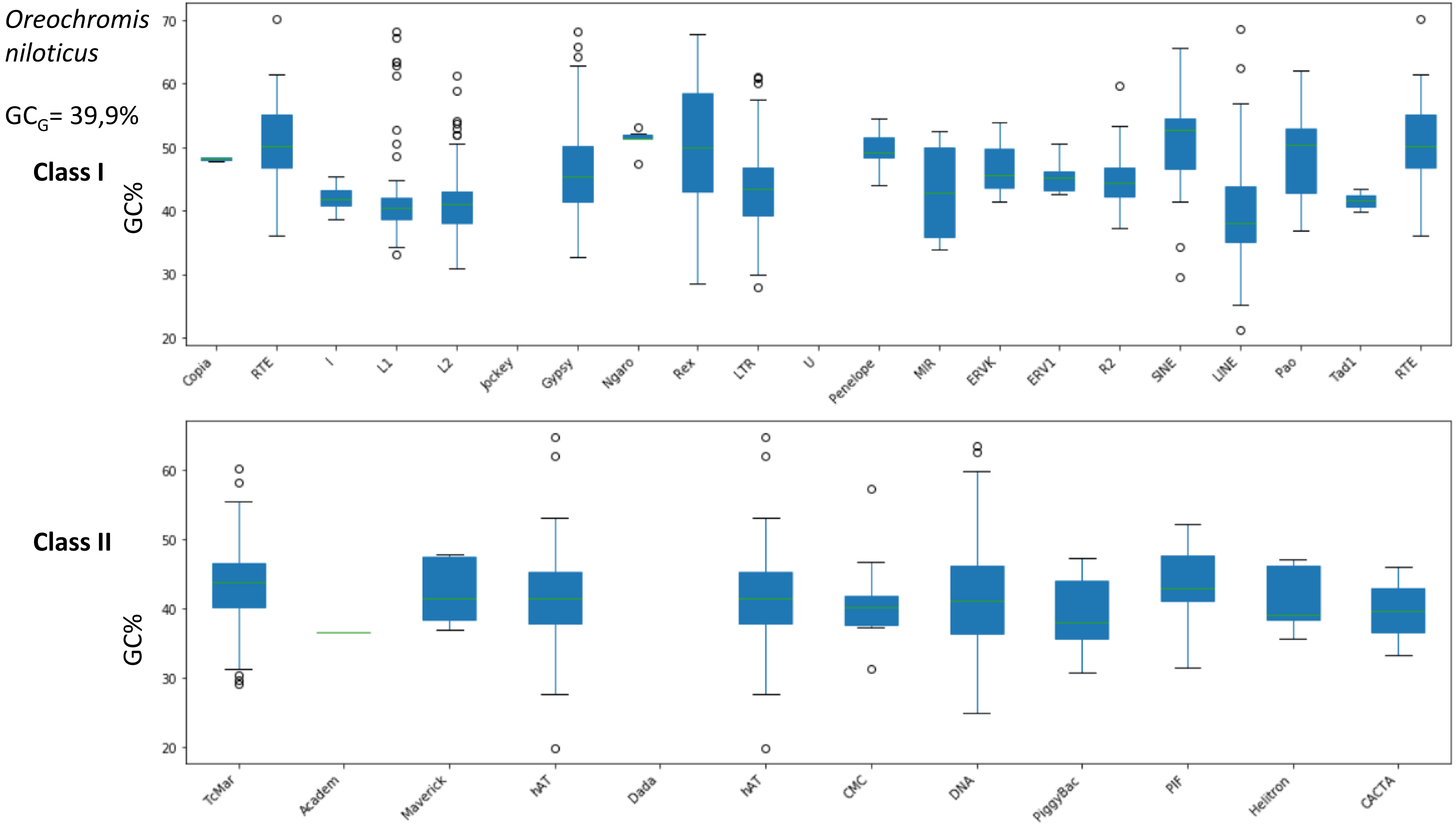

Oreochromis niloticus
GCG= 39,9%
Class I
GC%
70
60
50
40
30
20
Copia
RTE
I
L1
L2
Jockey
Gypsy
Ngaro
Rex
LTR
U
Penelope
MIR
ERVK
ERV1
R2
SINE
LINE
Pao
Tad1
RTE
Class II
GC%
60
50
40
30
20
TcMar
Academ
Maverick
hAT
Dada
hAT
CMC
DNA
PiggyBac
PIF
Helitron
CACTA

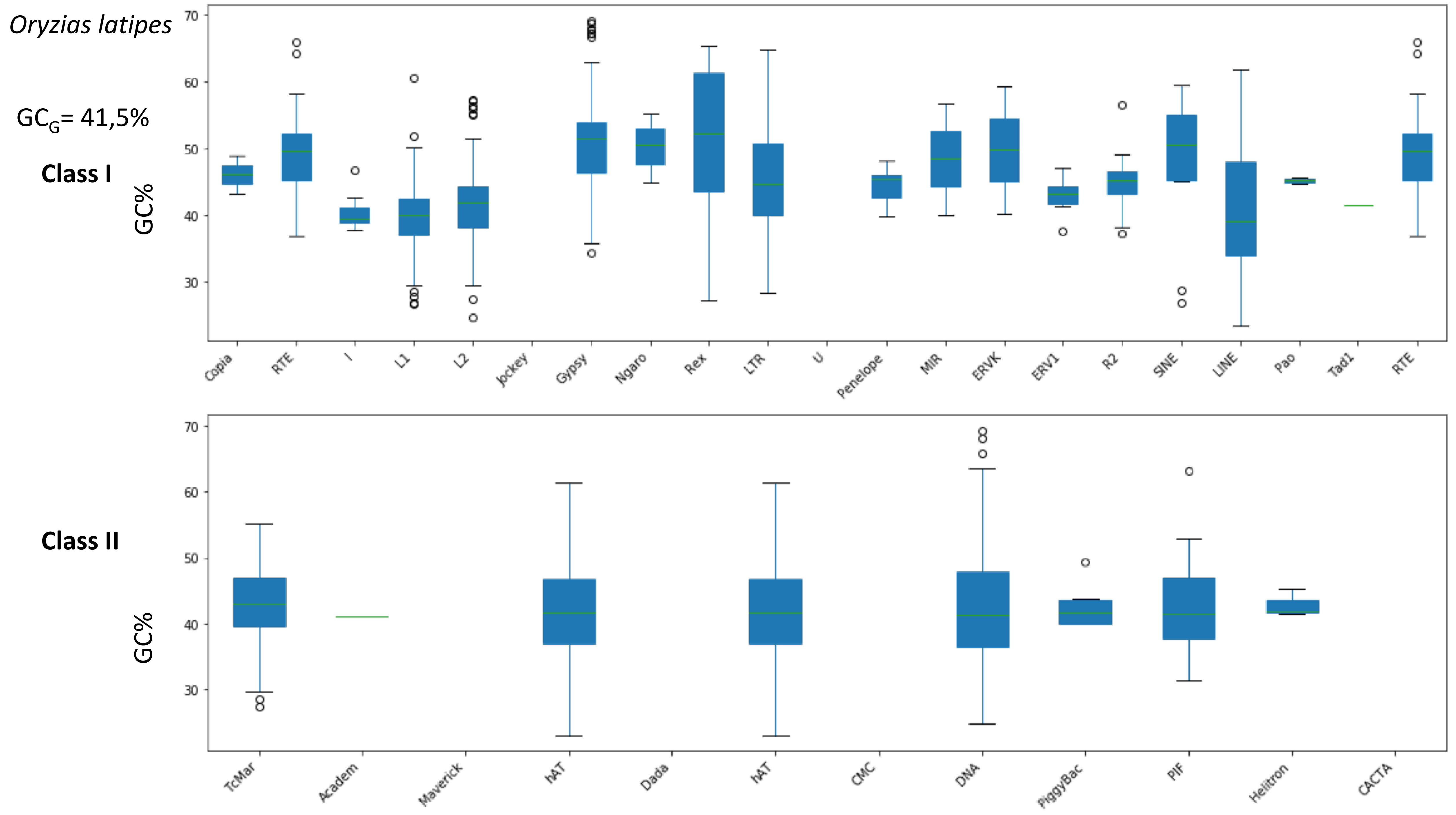
Oryzias latipes
GCG= 41,5%
Class I
Class II
GC%
GC%
70
60
50
40
30
Copia
RTE
I
L1
L2
Jockey
Gypsy
Ngaro
Rex
LTR
U
Penelope
MIR
ERVK
ERV1
R2
SINE
LINE
Pao
Tad1
RTE
TcMar
Academ
Maverick
hAT
Dada
hAT
CMC
DNA
PiggyBac
PIF
Helitron
CACTA

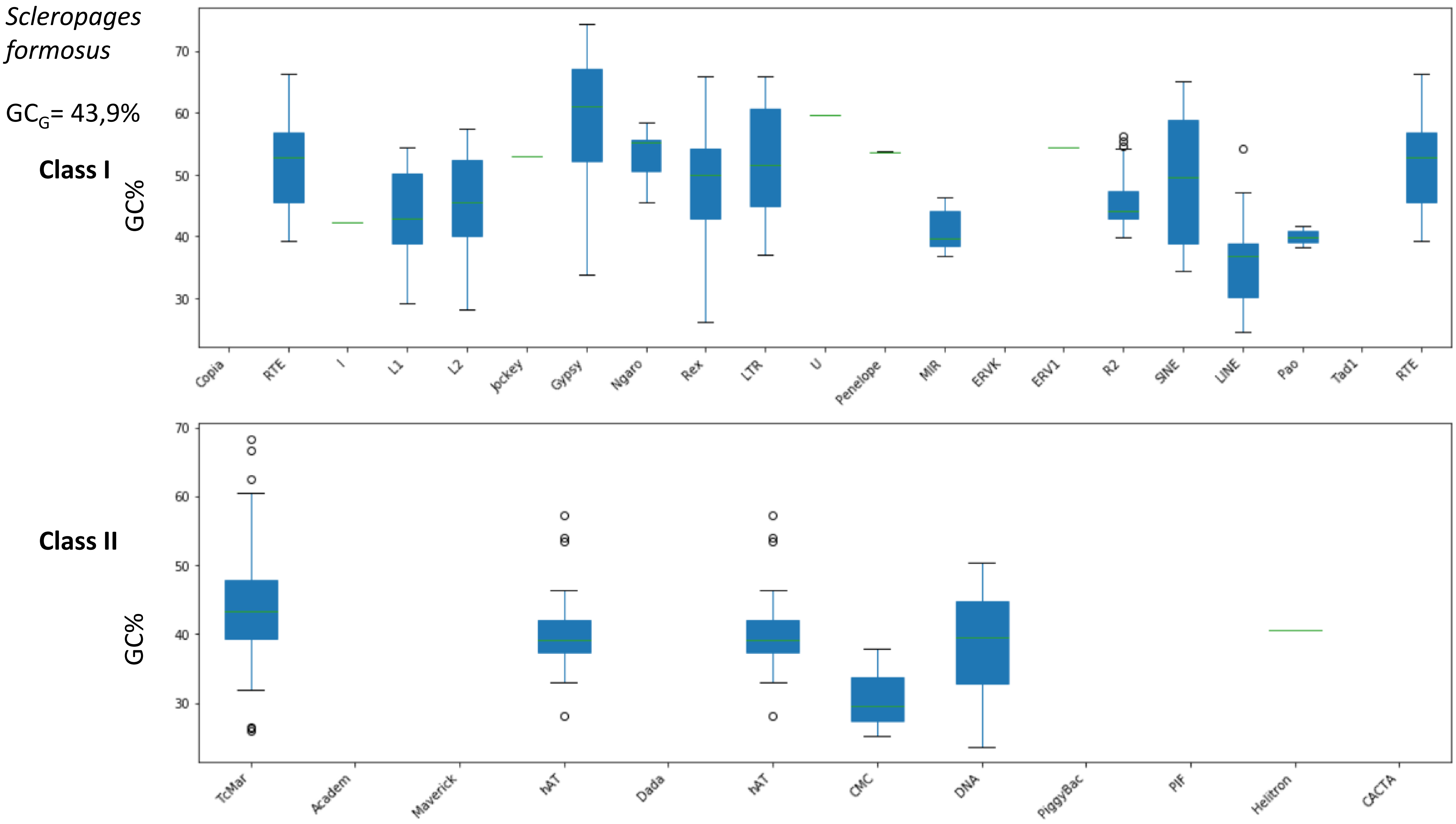

Scleropages formosus
GC_G= 43,9%
Class I
GC%
70
60
50
40
30
Copia
RTE
I
L1
L2
Jockey
Gypsy
Ngaro
Rex
LTR
U
Penelope
MIR
ERVK
ERV1
R2
SINE
LINE
Pao
Tad1
RTE
Class II
GC%
70
60
50
40
30
TcMar
Academ
Maverick
hAT
Dada
hAT
CMC
DNA
PiggyBac
PIF
Helitron
CACTA

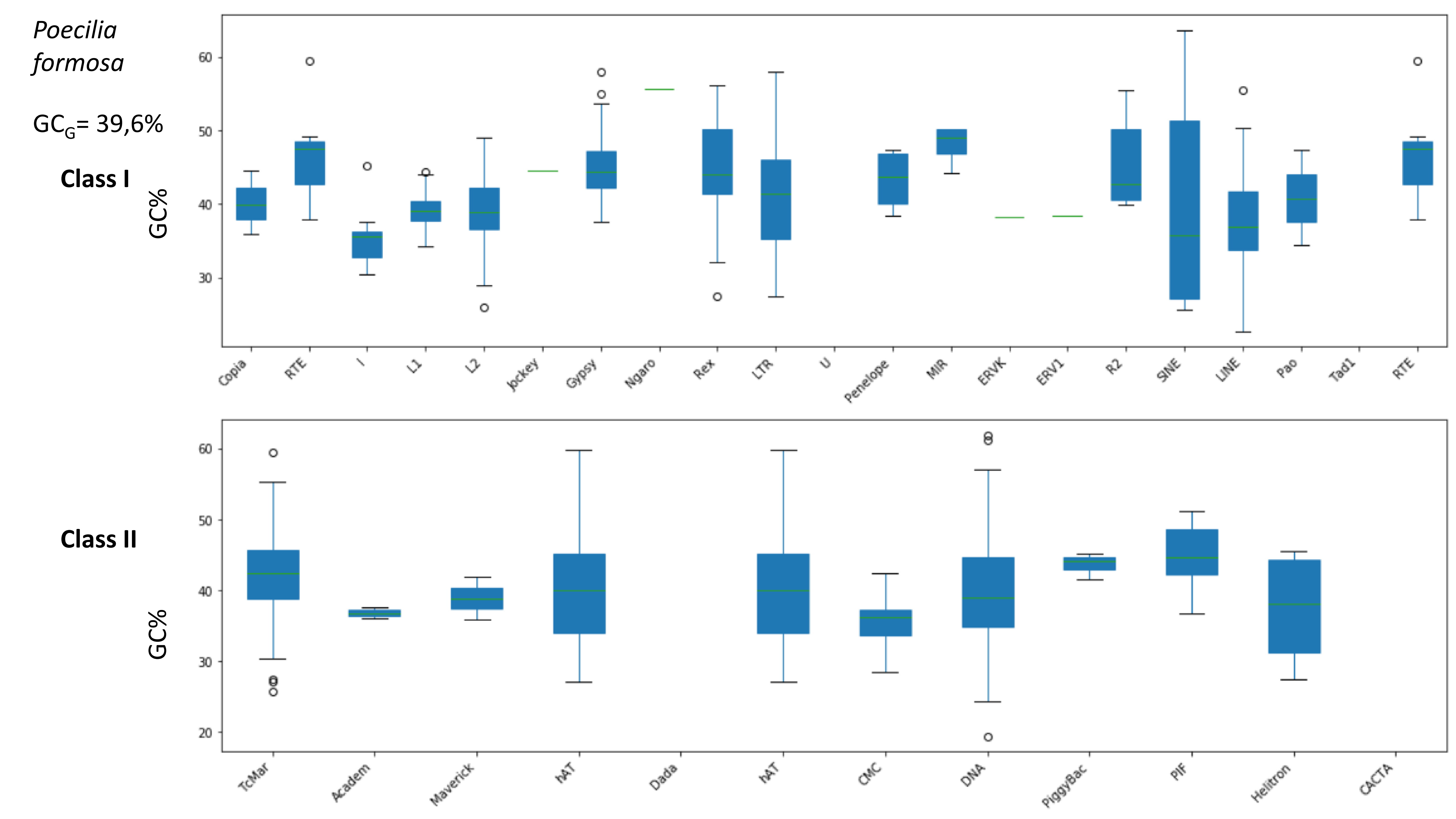
Poecilia formosa
GC_G= 39,6%
Class I
GC%
60
50
40
30
Copia
RTE
I
L1
L2
Jockey
Gypsy
Ngaro
Rex
LTR
U
Penelope
MIR
ERVK
ERV1
R2
SINE
LINE
Pao
Tad1
RTE
Class II
GC%
60
50
40
30
20
TcMar
Academ
Maverick
hAT
Dada
hAT
CMC
DNA
PiggyBac
PIF
Helitron
CACTA

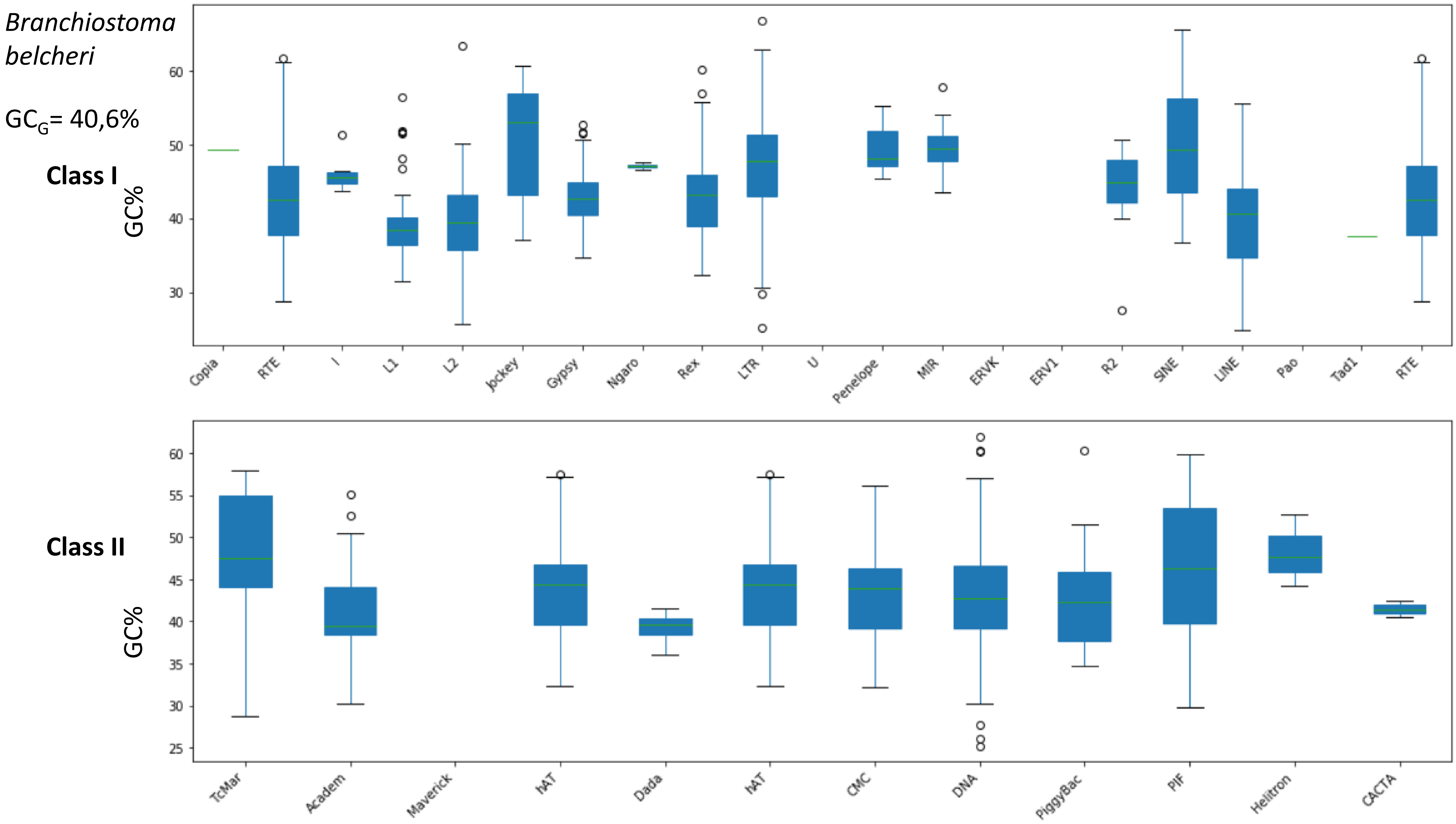

Branchiostoma belcheri
GC_G= 40,6%
Class I
GC%
60
50
40
30
Copia
RTE
I
L1
L2
Jockey
Gypsy
Ngaro
Rex
LTR
U
Penelope
MIR
ERVK
ERV1
R2
SINE
LINE
Pao
Tad1
RTE
Class II
GC%
60
55
50
45
40
35
30
25
TcMar
Academ
Maverick
hAT
Dada
hAT
CMC
DNA
PiggyBac
PIF
Helitron
CACTA

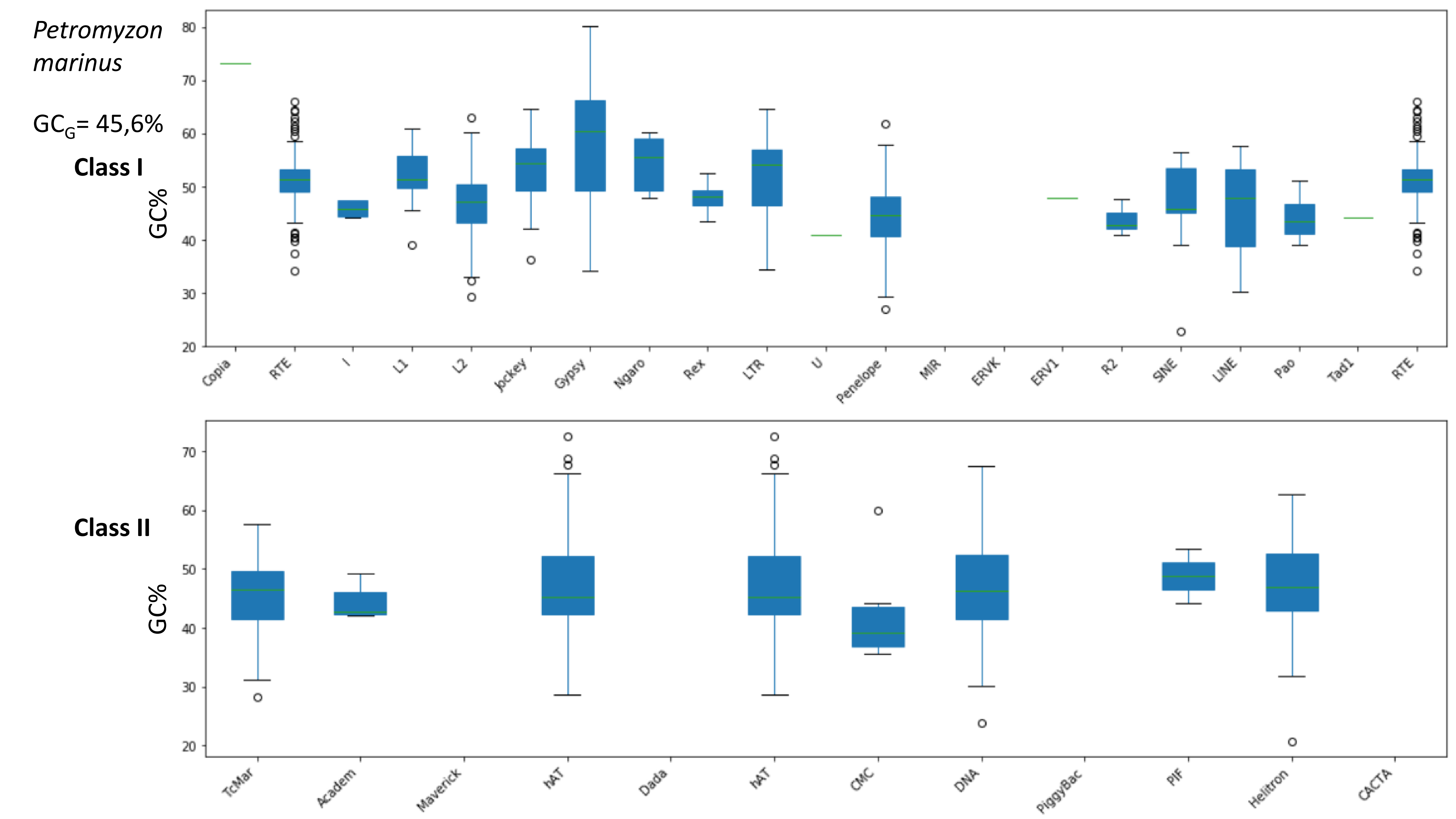

Petromyzon marinus
GCG= 45,6%
Class I
GC%
80
70
60
50
40
30
20
Copia
RTE
I
L1
L2
Jockey
Gypsy
Ngaro
Rex
LTR
U
Penelope
MIR
ERVK
ERV1
R2
SINE
LINE
Pao
Tad1
RTE
Class II
GC%
70
60
50
40
30
20
TcMar
Academ
Maverick
hAT
Dada
hAT
CMC
DNA
PiggyBac
PIF
Helitron
CACTA

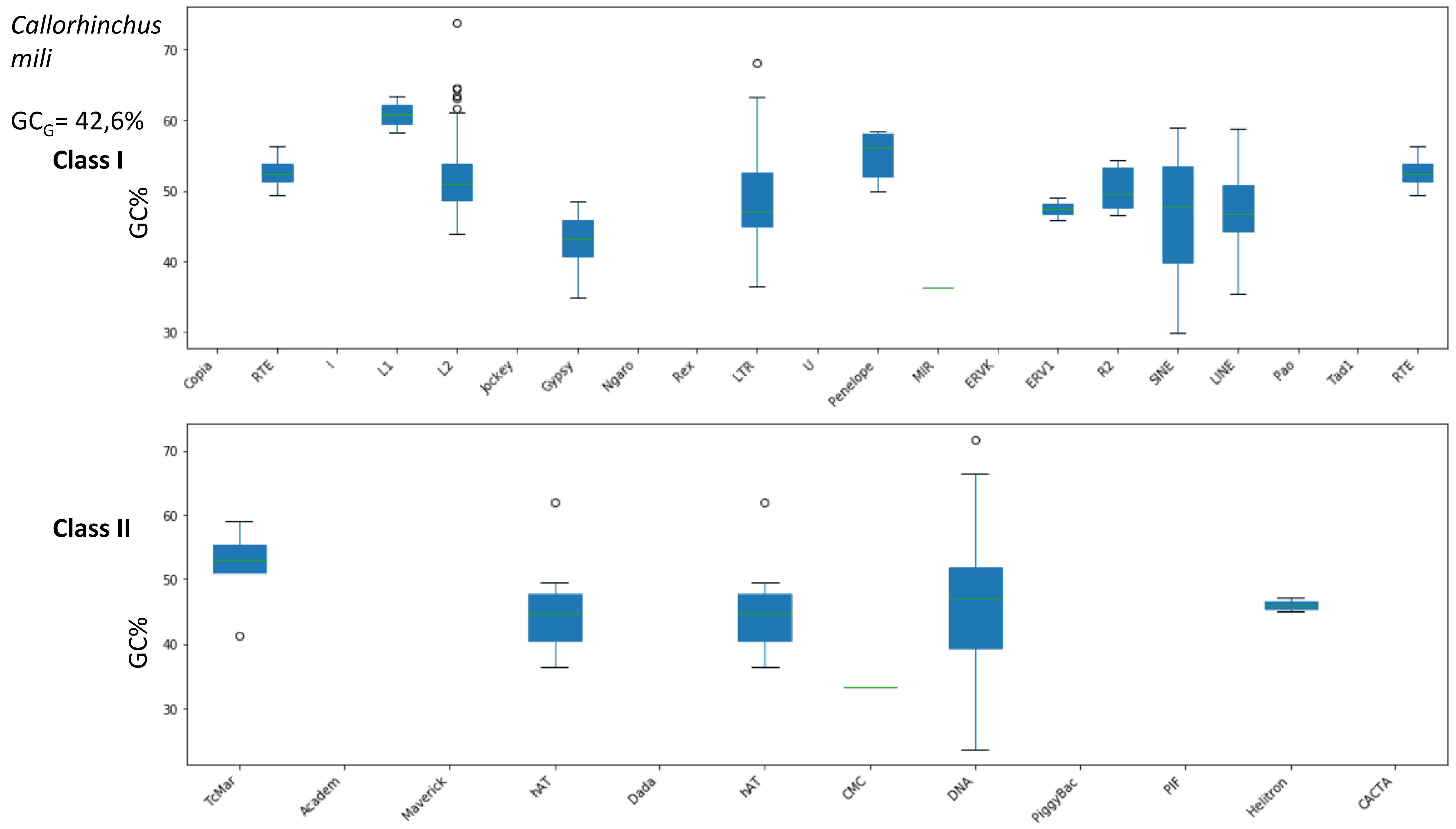
Callorhinchus mili
GC_G= 42,6%
Class I
GC%
70
60
50
40
30
Copia
RTE
I
L1
L2
Jockey
Gypsy
Ngaro
Rex
LTR
U
Penelope
MIR
ERVK
ERV1
R2
SINE
LINE
Pao
Tad1
RTE
Class II
GC%
70
60
50
40
30
TcMar
Academ
Maverick
hAT
Dada
hAT
CMC
DNA
PiggyBac
PIF
Helitron
CACTA
